# Oxygen Nonstoichiometry and Valence State of Manganese in La_1–*x*_Ca_*x*_MnO_3+δ_

**DOI:** 10.1021/acsomega.1c00208

**Published:** 2021-04-02

**Authors:** Sabrina A. Heuer, Roland Schierholz, Evgeny V. Alekseev, Lars Peters, David N. Mueller, Tomáš Duchoň, Vaibhav Vibhu, Hermann Tempel, Lambertus G. J. de Haart, Hans Kungl, Rüdiger-A. Eichel

**Affiliations:** † Institute of Energy and Climate Research (IEK-9), Forschungszentrum Jülich GmbH, Wilhelm-Johnen-Straße, DE-52425 Jülich, Germany; ‡ Institute of Physical Chemistry, RWTH Aachen University, Landoltweg 2, DE-52074 Aachen, Germany; § Institute of Crystallography, RWTH Aachen University, Jägerstraße 17-19, DE-52066 Aachen, Germany; ∥ Peter Grünberg Institute (PGI-6), Forschungszentrum Jülich GmbH, Wilhelm Johnen Straße, DE-52425 Jülich, Germany

## Abstract

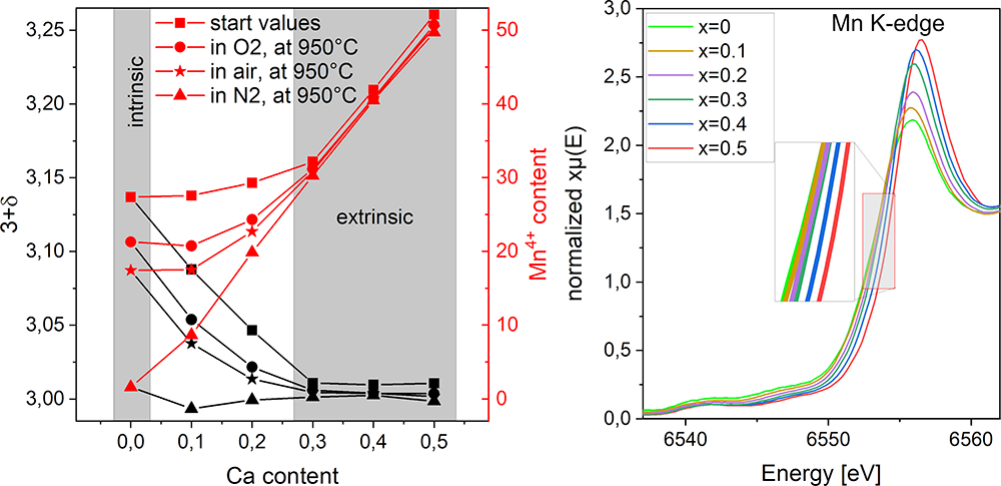

Perovskites of the ABO_3_ type, such as LaMnO_3_, can be used as air electrodes in solid oxide fuel cells and electrolyzers. Their properties can be tuned by A- and B-site substitutions. The influence of La substitution by Ca on the oxygen nonstoichiometry has been investigated frequently, but the results depend highly on the synthesis and atmospheric conditions. In this work, a series of La_1–*x*_Ca_*x*_MnO_3+δ_ (*x* = 0–0.5) was synthesized using conventional solid-state synthesis under an air atmosphere. The structures of the materials were studied in detail with powder X-ray diffraction. The initial oxygen nonstoichiometries were determined using thermogravimetric reduction. The samples were subsequently analyzed in terms of defect chemistry in dependence of temperature, atmosphere, and Ca content via thermogravimetric analysis. The changes in the manganese charge states were investigated by X-ray absorption near-edge spectroscopy experiments. The influence of intrinsic and extrinsic effects on the Mn-valence state of the differently Ca-substituted samples as calculated from thermogravimetric analysis and as determined directly from X-ray absorption near-edge spectroscopy is presented.

## Introduction

Lanthanum–manganese oxides are frequently discussed for application as oxygen electrodes, for example, in solid oxide cells, as catalysts or as oxygen sensors and supercapacitors.^1−5^ Some of them are also well known for their colossal magnetoresistive effect, such as Ca-doped LaMnO_3_.^6−8^ Substitutions at the lanthanum site, for example, by calcium, revealed the possibility to change the material structure and defect chemistry. Defect chemistry is mainly governed by oxygen nonstoichiometry and manganese charge state and plays an important role for the catalytic activity of the material.^9−12^ Material synthesis methods and conditions affect the defect chemistry.^12−17^ As they are exposed to varying atmospheres at high temperatures during their application, it is important to investigate how the defect chemistry changes under varying temperature and atmosphere. In contrast to the classical oxygen-deficient perovskites, such as La_1–*x*_A′_*x*_BO_3−δ_ (A′ = Ca or Sr and B = Fe or Co), LaMnO_3_ and its Ca-substituted derivatives are frequently described as oxygen-excess perovskites in the form of La_1–*x*_Ca_*x*_MnO_3+δ_. Investigations on the oxygen excess in La_1–*x*_Ca_*x*_MnO_3+δ_ have been performed, and it appeared that oxygen excess in La_1–*x*_Ca_*x*_MnO_3+δ_ is not possible by the introduction of oxygen into interstitial sites. Instead, it was found that A- and B-site cation vacancies in the form of (La, Ca)_1−ε_Mn_1−ε_O_3_ (ε = δ/(3 + δ)) are established.^14,18−20^ However, for comparability with the literature, in general, the oxygen excess is considered.

The material properties of the unsubstituted LaMnO_3+δ_ highly depend on its oxygen nonstoichiometry δ and the corresponding Mn-oxidation state.^18^ Previous structural studies suggested three different crystal structures for room temperature LaMnO_3+δ_. Stoichiometric LaMnO_3_ has the pseudocubic O′-orthorhombic structure (, *Pnma*) and can transform into a less distorted O-orthorhombic structure.^12,18,21^ Bogush et al.^21^ defined  (*Pnma*) for this O-phase, while Dabrowski et al.^12^ assumed  The transition into a rhombohedral structure (*R*3̅*c*) is also possible.^21^

The perovskite structures are mainly dependent on the relative equilibrium bond lengths within the perovskite. The Goldschmidt tolerance factor gives an indication to which extent the bond lengths (B–O) and (A–O) deviate from the ideal cubic perovskite with the A-site cation on (0 0 0) and the B-site cation on (1/2 1/2 1/2).^17,22^ For a perovskite under ambient conditions, it can be calculated using the ionic radii (e.g., after Shannon^23^) as presented in the following eq 1.
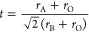
1

For the cubic perovskites, the Goldschmidt tolerance factor is 1. Deviations represent a decrease in the symmetry of the materials. In unsubstituted LaMnO_3_, the Goldschmidt tolerance factor is *t* = 0.954, assuming a coordination number of 12 for La and a coordination number of 6 for all other ions.^18^ With increasing Mn^4+^ content, the Goldschmidt tolerance factor increases, as pointed out by Töpfer and Goodenough.^18^ An increase in the Mn^4+^ concentration within LaMnO_3_ can be obtained intrinsically by influencing the oxygen stoichiometry and the formation of defects or extrinsically by A-site substitution, for example, with Ca.

Starting with 100% Mn^3+^ as in LaMnO_3_, the MnO_6_ octahedra are strongly Jahn–Teller-distorted and three different Mn–O bond lengths can be found. This results in the strongly distorted O′-phase with  as a result of the superposition of the Jahn–Teller distortion with the orthorhombic distortion introduced by the tolerance factor t < 1.^12,18,21^ This distortion can be reduced by the incorporation of oxygen (LaMnO_3+δ_), which is accompanied by an increasing Mn^4+^ content. At about 14% Mn^4+^, the Jahn–Teller distortion disappears and only one Mn–O bond length is present within the MnO_6_ octahedra. In this O-phase, the orthorhombic distortion is only induced by the tolerance factor *t* < 1 and the octahedra are tilted around the *a* and *b* axis.^12,18,21^ With even higher Mn^4+^ contents (20–22%), the structure becomes rhombohedral (R-phase) and the octahedra are tilted around the cubic ⟨111⟩ directions.^21,24^

**Scheme 1:**
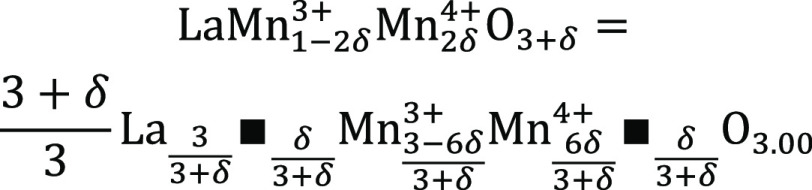
Mixed Oxidation State Structure in LaMnO_3_

The interrelation between the Mn-oxidation state and oxygen stoichiometry has been explained by the establishment of mixed oxidation state structures (Mn^3+^/Mn^4+^) within LaMnO_3+δ_, as shown in Scheme 1.^18^ According to this, the Mn^4+^ content is correlated with 2δ.

Black squares ■ represent the cation vacancies on the A- and B-sites. Oxygen uptake (change in the δ-value) and simultaneously partial change in the Mn-oxidation state from Mn^3+^ to Mn^4+^ lead to a reduced distortion. These changes within stoichiometric LaMnO_3_ are not influenced by substitution, and as such, we define them as intrinsic. Töpfer et al.^18,25^ described that if δ < 0.1, the structure is O′-orthorhombic, while it is rhombohedral for δ > 0.1. They observed only the O′- and the R-structure for LaMnO_3+δ_ at room temperature. The transition via the O-phase for intermediate δ-values (0.1 ≤ δ ≤ 0.15) was only reported for temperatures below room temperature by Bogush et al. contrary to Dabrowski et al., who report this phase for 0.05 ≤ δ ≤ 0.1 at room temperature.^12,20^

Substituting LaMnO_3_ with divalent cations, such as Ca^2+^, leads to an extrinsically controlled transition of Mn^3+^ to Mn^4+^, which stabilizes the structure from O′-orthorhombic to O-orthorhombic due to increasing Mn^4+^ content.^12^ Dabrowski et al.^12^ showed that the O′-phase is stable only for very small δ-values close to zero and calcium contents *x* < 0.15.

**Scheme 2:**
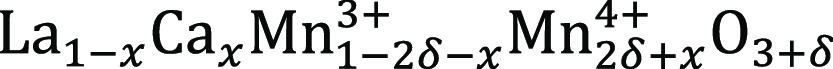
Mixed Oxidation State in La_1–*x*_Ca_*x*_MnO_3+δ_

Similar to Scheme 1 (without cation vacancies), the Mn-oxidation state of the calcium substituted samples can be calculated as presented in Scheme 2.

In this, the influence of both, the change in the δ-value and the introduction of Ca^2+^ (leading to a forced transition of Mn^3+^ to Mn^4+^), on the Mn^4+^ content is considered.

Scheme 2 stresses the importance of experimentally derived δ-values for the determination of the Mn^4+^ content. In this work, we present the results of the systematic investigation of the La_1–*x*_Ca_*x*_MnO_3+δ_ system with respect to the changes in its oxygen stoichiometry and Mn-oxidation state in dependence of Ca content, atmosphere, and temperature by powder X-ray diffraction (PXRD), thermogravimetric analysis (TGA), and X-ray absorption near edge spectroscopy (XANES).

## Experimental Details

### Material Preparation

La_1–*x*_Ca_*x*_MnO_3+δ_ (*x* = 0–0.5) was synthesized via conventional solid-state synthesis. La_2_O_3_ (Alfa Aesar, 99.99%) was dried for 12 h at 850 °C before usage. Stoichiometric mixtures of the as-prepared La_2_O_3_, CaCO_3_ (Alfa Aesar, 99.9%), and MnO_2_ (Alfa Aesar, 99.9%) were ball-milled with isopropyl alcohol for 4 h. The dried powders were calcined in air for 8 h at 1100 °C and ground to their final state with a pestle and mortar. Some of the as-prepared LaMnO_3+δ_ and La_0.9_Ca_0.1_MnO_3+δ_ powders were additionally tempered for 4 h at 1000 °C in a nitrogen atmosphere.

### Powder X-ray Diffraction and Rietveld Refinement

Qualitative characterization of the calcined powders was performed with PXRD on a PANalytical Empyrean Series 2 diffractometer equipped with a PANalytical X’Celerator detector, using a Cu Kα radiation source. The PXRD measurements for Rietveld refinement were performed using a Bruker AXS D8 Advance powder diffractometer with Cu Kα radiation (λ_1_ = 154.0596 pm and λ_2_ = 154.4493 pm) equipped with a LynxEye semiconductor strip detector (SSD) and Ni filter. θ/θ scans were performed for each sample in three ranges in steps of Δ2θ = 0.0105°: (i) 10–60° 2θ, counting 0.8 s per step, (ii) 60–100° 2θ, counting 1.7 s per step, and (iii) 100–140° 2θ, counting 3.5 s per step, summing up to about 7 h scanning time per sample. Powders were prepared onto flat sample holders. Structure refinements with the Rietveld method^26^ were performed using the TOPAS Academic suite.^27^ The refinements were carried out simultaneously against all three measurement ranges taken for each of the compounds. A fundamental parameter approach was used to calculate the line profiles.^28^ The lattice parameters of the perovskite-type phases, the fractional coordinates of the atoms (for those coordinates allowed to vary by symmetry), isotropic thermal displacement parameters for each element (or the site for mixed occupancies), the sample height, and size and strain contributions to the peak shape were refined simultaneously against all ranges as “global” parameters. A 6- to 10-coefficient background polynomial, a scaling factor, and a minor Cu Kβ-contamination were refined individually for the ranges.

### Determination of the Initial Oxygen Nonstoichiometry

**Scheme 3:**
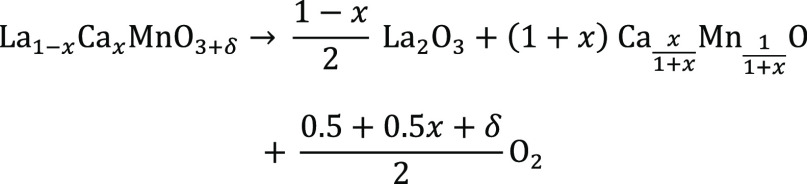
Decomposition reaction for thermogravimetric reduction

The initial oxygen content in the studied materials was determined using a thermogravimetric analyzer (TGA 5500 from TA Instruments). The powdered sample (50 mg) was weighed into a platinum crucible. Pressurized air (99.999%) and forming gas (99.1%) consisting of argon and 4% hydrogen have been used for TGA experiments. The calcined materials were heated twice to 950 °C (5 K/min) in a pressurized air atmosphere and then cooled (5 K/min) down, in order to stabilize the system. In an Ar/4%H_2_ flow, the sample was slowly heated to 900 °C with a rate of 0.5 K/min. Mass changes were continuously recorded, and the final masses after reduction (under Ar/4%H_2_ atmosphere) were taken for calculation of the initial oxygen nonstoichiometry (eq 2). The materials are decomposed upon reduction as shown in Scheme 3. The decomposition into such a mixed structure was verified by PXRD (see Figure S2). As presented in Scheme 3, the knowledge of the decomposition products allows the calculation of the samples’ initial delta values via their mass loss upon reduction according to eq 2. It also shows that the oxygen content in the final solid products is lower than that in La_1–*x*_Ca_*x*_MnO_3+δ_.
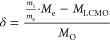
2here *m*_i_ describes the initial sample mass of La_1–*x*_Ca_*x*_MnO_3+δ_, *m*_e_ is the end mass of the decomposition products, *M*_e_ is the molar mass of the decomposition products, *M*_LCMO_ is the molar mass of La_1–*x*_Ca_*x*_MnO_3.00_, and *M*_O_ is the molar mass of oxygen.

### Determination of Changes in Oxygen Nonstoichiometry in Various Atmospheres and Temperatures

Air-calcined La_1–*x*_Ca_*x*_MnO_3+δ_ (*x* = 0–0.5) and additionally nitrogen-tempered LaMnO_3_ and La_0.9_Ca_0.1_MnO_3+δ_ were measured in oxygen (99.9992%), nitrogen (99.9992%), and synthetic air (80% N_2_, 20% O_2_) in a thermogravimetric analyzer (STA 449 F1 Jupiter from Netzsch) to determine changes in their oxygen stoichiometry. An aluminum oxide crucible containing 500 mg of the sample was used for these measurements. For buoyancy correction, measurements were conducted on empty crucibles for all applied gases and temperatures. Two cycles were performed: in each, the samples were heated with 5 K/min to 950 °C, held for 5 h, and cooled down with the same rate to room temperature. In the first cycle, the desired atmosphere is applied to the air-calcined samples. Reversibility of the data obtained after cycle 1 was verified by performing a second measurement cycle. Experiments with a third cycle (not shown) revealed that the second cycle already delivers reversible results. With the help of the previously determined initial δ-values via reduction, the measured mass changes could be correlated with the samples’ changes in their δ-value and, therefore, changes in their oxygen nonstoichiometry, which in the following will be written as 3+δ.

Temperature-dependent TGA of La_0.9_Ca_0.1_MnO_3+δ_ was performed in O_2_ to determine whether the sample is in equilibrium when heated up to 950 °C. Therefore, air-calcined La_0.9_Ca_0.1_MnO_3+δ_ was investigated from 550 to 950 °C in 100 °C steps. As described before, two cycles with a 5 h holding time at the respective temperature were performed. The resulting mass changes were correlated to the samples’ specific oxygen nonstoichiometry.

The same two cycles up to 950 °C were performed for LaMnO_3_ that was either calcined in air or additionally tempered in N_2_ before measurement. These measurements were performed in O_2_, N_2_, and synthetic air.

### Investigation of the Mn Electronic State Using XANES

Mn K-edge X-ray absorption spectra were recorded at beamline P65 at PETRA III, Deutsches Elektronen-Synchrotron (DESY) in Hamburg, Germany.^29^ The undulator X-ray beam is focused using a water-cooled fixed exit Si double-crystal monochromator, using the Si 111 lattice plane. In order to avoid contributions from higher harmonics, two plane mirrors with variable angles of incidence are placed in front of the monochromator. For the measurements, the powdered samples were mixed with appropriate amounts of cellulose powder and pressed into thin pellets, which were placed in a sample holder that is mounted in the beam path. Absorption spectra were recorded in transmission geometry, with the first ionization chamber placed in front of the sample collecting incident beam intensity and a second ionization chamber collecting sample intensity to yield the absorption coefficient μ(*E*).

The photon energy used for measurements ranged from 6400 to 7500 eV. The spectra were normalized using a linear pre-edge and a second-order polynomic post-edge utilizing the program Athena.^30^

O K-edge and Mn L_32_-edge spectra were collected on powdered samples at the UE56/1-SGM beamline at Helmholtz-Zentrum Berlin, in the total electron yield detection mode. Spectra are two-point normalized to the post- and pre-edge.

## Results and Discussion

### Powder X-ray Diffraction

**Figure 1:**
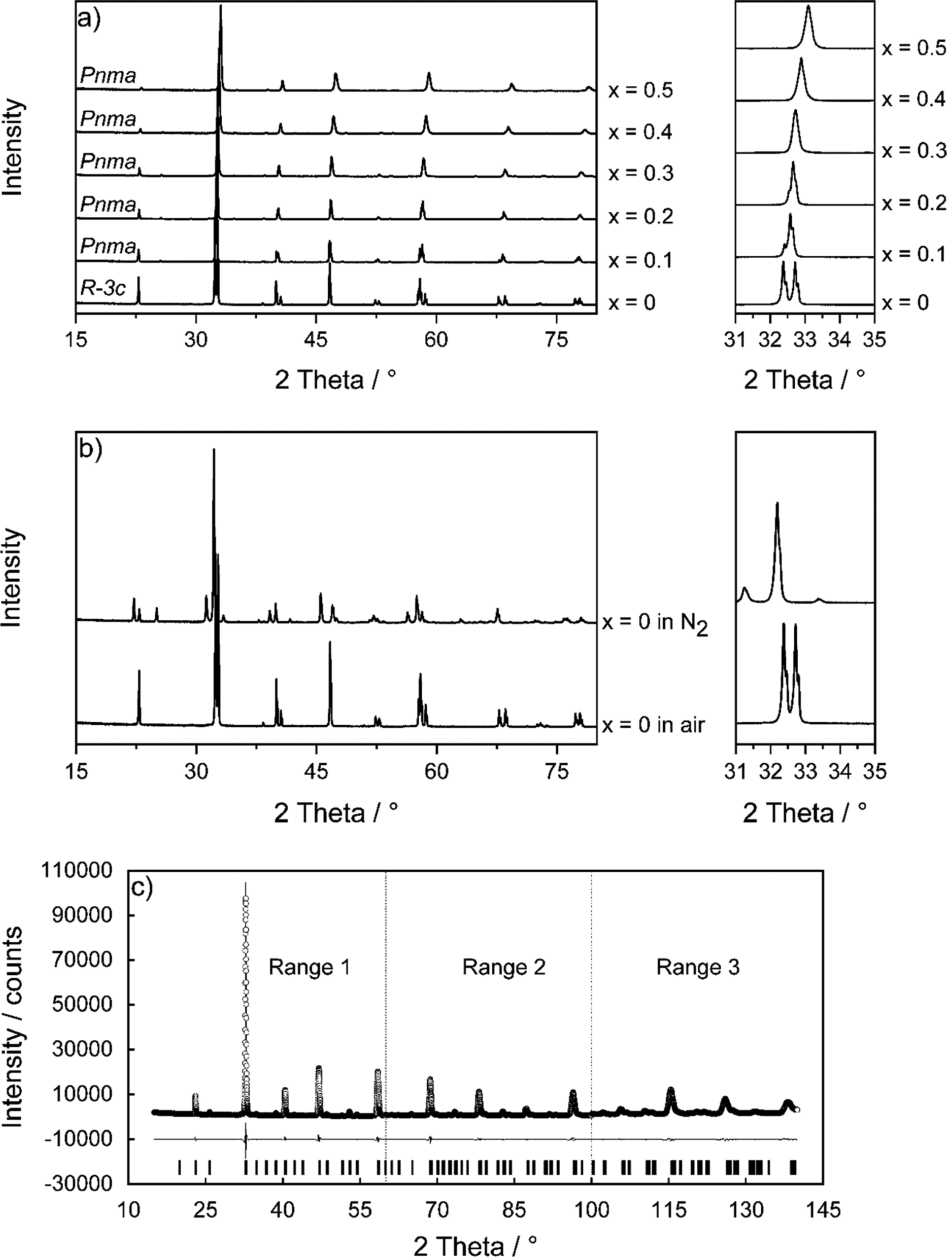
Normalized PXRD patterns of (a) Ca-substituted La_1–*x*_Ca_*x*_MnO_3+δ_ (*x* = 0–0.5) synthesized in air, including a detailed graphic of the main reflection. (b) LaMnO_3_ synthesized in air and after tempering in N_2_, including a graphic of the main reflection. Individual diffractograms are shown with a constant but arbitrary intensity offsets for the sake of visibility. (c) Plot of the refinement results using the Rietveld method for La_0.7_Ca_0.3_MnO_3+δ_. Circles represent observed intensities, and the solid line represents calculated intensities. The gray line is the intensity difference curve. Small vertical tic marks indicate the reflection positions of the perovskite-type phase. Dotted vertical lines indicate the three measurement ranges.

PXRD measurements of the Ca-substituted La_1–*x*_Ca_*x*_MnO_3+δ_ (*x* = 0–0.5) series synthesized at 1100 °C in air are shown in Figure 1a. They indicate a change in the crystal structure from a rhombohedral *R*3̅*c* structure of the unsubstituted LaMnO_3+δ_ sample to an orthorhombic structure *Pnma* of the Ca-substituted samples. LaMnO_3+δ_ shows a change in the space group to orthorhombic *Pnma* when tempered for 4 h at 1000 °C in a nitrogen atmosphere (Figure 1b).

Comparison of LaMnO_3_ and La_1–*x*_Ca_*x*_MnO_3+δ_ reveals a shift of the main reflection position toward higher angles with increasing Ca content. This indicates that the unit cell volume shrinks on substituting La^3+^ by Ca^2+^. This is crystal-chemically explainable, as the ionic radius of Ca^2+^ (134 pm) is slightly smaller than that of La^3+^ (136 pm).^23^ In addition to this, the increasing oxidation of Mn^3+^ to Mn^4+^ and the resulting changes toward smaller ionic radii are expected to bias the lattice parameters as well.^31,32^

**Figure 2:**
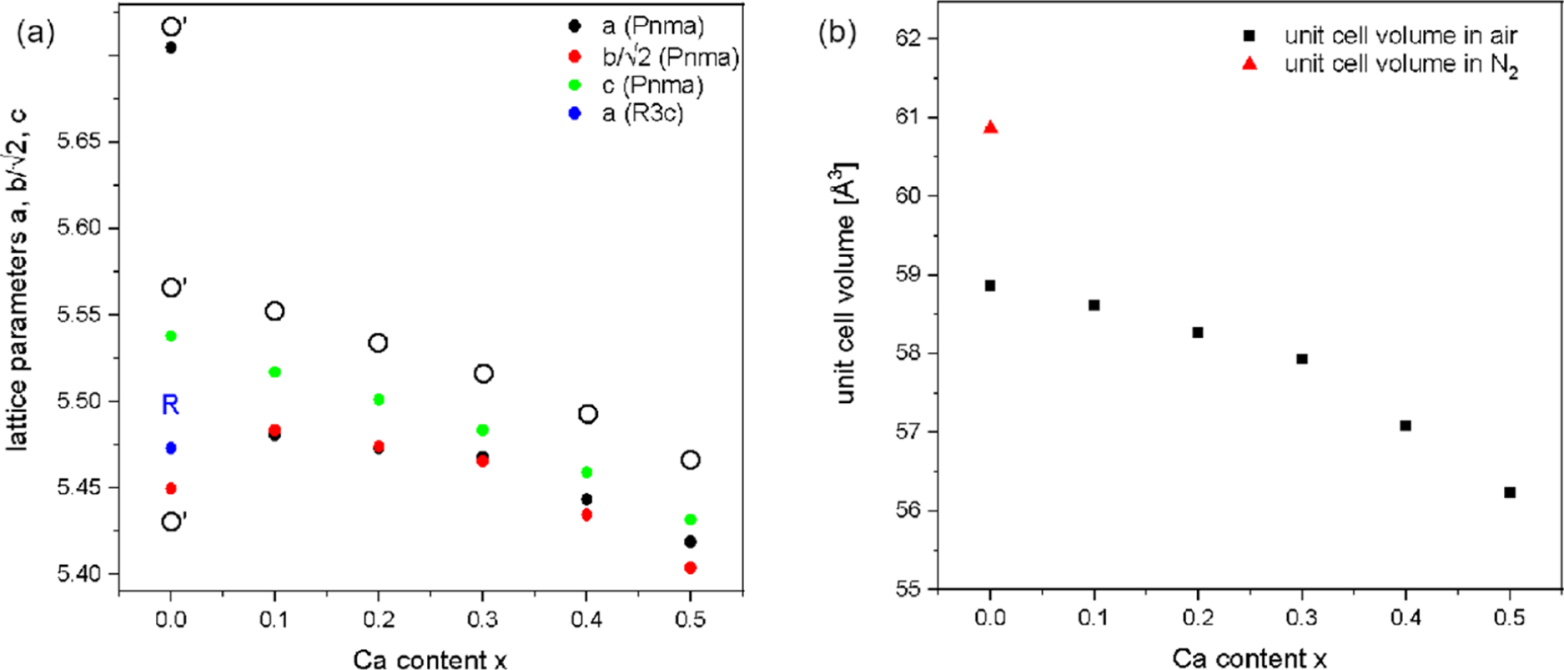
(a) Lattice parameters *a*, , and *c* for the orthorhombic samples and *a*_trigonal_ (α = 60.69°) for the rhombohedral cell as determined by PXRD and a corresponding Rietveld refinement of the La_1–*x*_Ca_*x*_MnO_3+δ_ series. This shows that all samples except for LaMnO_3+δ_ are of the orthorhombic O structure with . For LaMnO_3+δ_, either the rhombohedral structure is observed if synthesized in air or the strongly distorted O′-structure with  is present when treated in a N_2_ atmosphere. In (b), the calculated unit cell volumes of the differently Ca-substituted La_1–*x*_Ca_*x*_MnO_3+δ_ samples are shown.

Figure 1c shows an exemplary refinement of La_0.7_Ca_0.3_MnO_3+δ_ using the Rietveld method. Rietveld refinements were performed in detail against all diffraction data shown in Figure S1a. The obtained lattice parameters and unit cell volumes are presented in Table S1 and Figure 2a,b. They show that almost all samples are present in the less distorted O-phase with .^12^ Only pure LaMnO_3+δ_ is either rhombohedral R when calcined in air (δ = 0.137, Table 2) or in the strongly distorted O′-phase with  when calcined in nitrogen (δ = 0.076, Table 2). This agrees well with the values published by Töpfer et al.,^18,25^ who state that at room temperature, the O′-phase is stable for 0 ≤ δ ≤ 0.11 and the rhombohedral phase for δ ≥ 0.11. Bogush et al.^21^ find similar conditions for the rhombohedral phase (Mn^4+^ ≥ 22%, δ ≥ 0.11), but they found the O-phase for 0.07 ≤ δ ≤ 0.11 and the O′-phase for 0.00 ≤ δ ≤ 0.07. The table shown by Dabrowski et al.^12^ gives similar values for pure LaMnO_3+δ_ (R for δ ≥ 0.11, O for 0.04 ≤ δ ≤ 0.11, and O′ for 0.00 ≤ δ ≤ 0.04).

The unit cell volume of rhombohedral LaMnO_3+δ_, synthesized in air, is comparable to that of La_0.9_Ca_0.1_MnO_3+δ_. When LaMnO_3+δ_ is tempered in nitrogen, the unit cell volume increases drastically (>0.3%) from 58.8582(4) to 60.8625(8) Å^3^. According to the literature, LaMnO_3_ experiences significant structural deformation in a nitrogen atmosphere, as the amount of Mn^3+^ in the structure increases, leading to stronger Jahn–Teller-distortion.^16^ This leads to a highly distorted structure with a larger cell volume (orthorhombic). When synthesized in air, Mn^3+^ is increasingly oxidized to Mn^4+^, which is not as affected by Jahn–Teller distortions. This diminishes the distortion within the structures in accordance with the formation of a rhombohedral structure, which exhibits a smaller unit cell volume. The shrinkage of the unit cell has been explained by Miyoshi et al.^31,33,34^ as a result of oxygen incorporation, which indeed occurs by the formation of additional unit cells and cation vacancies. The latter is compensated by the transition of Mn^3+^ to Mn^4+^ which also reduces the cationic radius. With increasing Ca content, a decrease in the lattice parameters *a*, *b*, and *c* is observed. This trend corresponds to a shrinkage of the cell volume with increasing Ca content and has also been reported in the literature by Sagdeo et al.^32^

The explained and discussed trends from Table S1 are presented in Figure 2, where Figure 2a shows the changes in cell parameters of the *Pnma* structures of La_1–*x*_Ca_*x*_MnO_3+δ_, while Figure 2b contains the unit cell volumes of all air-synthesized and all nitrogen-treated samples. The orthorhombic lattice parameters follow a clear trend for 0.1 ≤ *x* ≤ 0.5 with . For LaMnO_3+δ_, a visible difference appears. While *c* still follows the trend as before,  decreases and *a* increases, and therefore, the structural transition to the O′-phase with  can be observed for the sample treated in N_2_. The air-calcined sample is rhombohedral with the trigonal lattice parameter *a* (α = 60.68°) being slightly smaller than all orthorhombic parameters for La_0.9_Ca_0.1_MnO_3+δ_.

The formation of cation vacancies, as well as Ca substitution, also affects the Goldschmidt tolerance factor. To determine this factor, the exact stoichiometry of La_1–*x*_Ca_*x*_MnO_3_ needs to be determined. Therefore, oxygen excess obtained from the TGA was converted into cation defects, and the corresponding quantity of Mn^3+^ and Mn^4+^ was determined, as described above in Scheme 1. The as-calculated contents of La, Ca, Mn, and O within the compositions were used for the Goldschmidt factor calculations. The ionic radii of the cation vacancies themselves were not explicitly included. The results are shown in Table 1.

**Table 1:** Calculation of the Goldschmidt Tolerance factors (*t*) of Air-Synthesized La_1–*x*_Ca_*x*_MnO_3+δ_ (*x* = 0–0.5) and Additionally N_2_-Treated LaMnO_3+δ_

*x*	0	0.1	0.2	0.3	0.4	0.5
*T*	0.958 (O′) 0.961 (R)	0.963	0.966	0.969	0.974	0.979

For the air-synthesized samples, an increasing Goldschmidt tolerance factor with increasing Ca substitution is observed.

The Goldschmidt tolerance factor ranges from *t* = 0.961 (*x* = 0) to *t* = 0.979 (*x* = 0.5), thus moving closer to the ideal cubic value (*t* = 1). This hints at an increasing symmetry and stability of the crystal structures with higher Ca contents. If the air-synthesized LaMnO_3+δ_ is treated additionally in N_2_, the Goldschmidt tolerance factor is calculated to be 0.958 (*x* = 0, N_2_), showing a decreased symmetry within this sample. This transition from the O′- to R-structure with an increasing tolerance factor for pure LaMnO_3+δ_ has already been postulated by Töpfer and Goodenough.^18^

### Calculation of the Oxygen Nonstoichiometry Using Thermogravimetry under a Reducing Atmosphere

Thermogravimetric reduction of La_1–*x*_Ca_*x*_MnO_3+δ_ (*x* = 0.1–0.5) in an Ar/4%H_2_ atmosphere allows for the determination of the initial oxygen nonstoichiometries of the samples.

The reduction of the materials within TGA experiments delivers the start mass (*m*_i_) and end mass (*m*_e_) of the samples before and after reduction and therefore is the basis for the calculation of δ.

**Figure 3:**
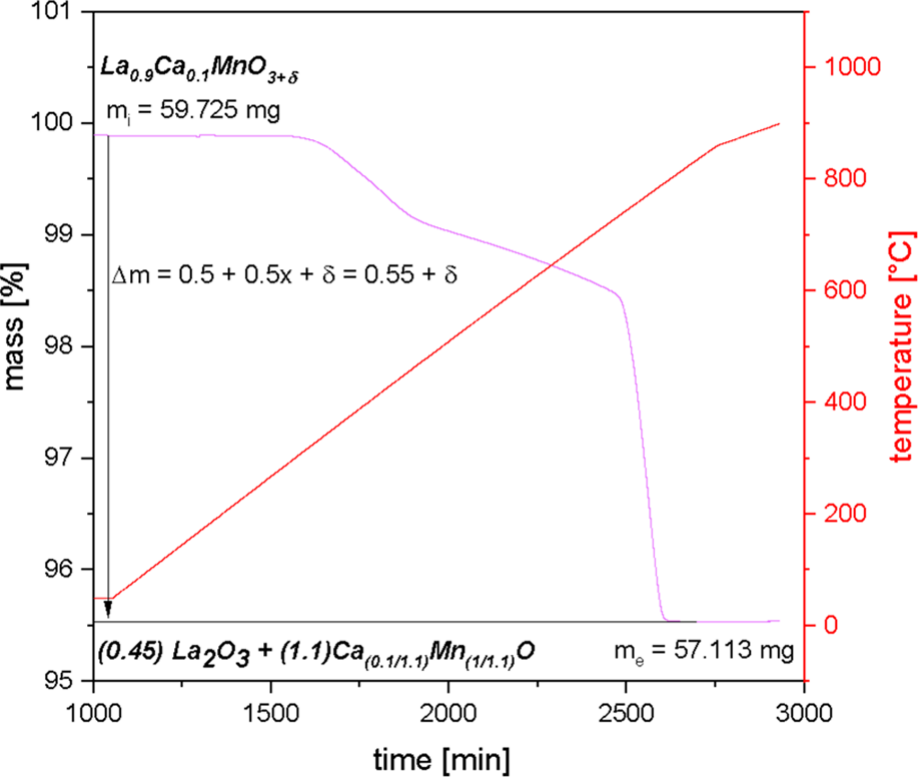
Mass change upon reduction of La_0.9_Ca_0.1_MnO_3+δ_ in an Ar/4%H_2_ atmosphere obtained from TGA. The sample is heated to 900 °C in a reducing atmosphere, where it is decomposed into its decomposition products and oxygen. The obtained mass difference is assumed to result from oxygen release. From this, the amount of oxygen can be calculated to determine the δ-value of the sample La_0.9_Ca_0.1_MnO_3+δ_.

It is assumed that only oxygen is released from the sample, and thus, the mass difference is only caused by oxygen. An example of how the δ-value is calculated from the mass loss measured during reduction of La_0.9_Ca_0.1_MnO_3+δ_ is shown in Figure 3, in which, according to Scheme 3, the amount of  of O_2_ is formed.

From the relative end mass *m*_e_/*m*_i_ (Table 2), the molar mass of stoichiometric La_0.9_Ca_0.1_MnO_3_, and the molar mass of its decomposition products (La_2_O_3_ and Ca_0.1/1.1_Mn_1/1.1_O), the respective δ-value can be calculated using eq 2. The decomposition products were characterized by PXRD measurements, as shown in Figure S2. For the specific Ca contents, the decomposition products are assumed to form according to Scheme 3. In the case of La_0.9_Ca_0.1_MnO_3_, a value of δ = 0.088 has been determined. The reduction of all air-synthesized samples La_1–*x*_Ca_*x*_MnO_3+δ_ (*x* = 0.1–0.5) is shown in Figure S3. The corresponding relative end masses and the calculated δ-values are listed in Table 2.

**Table 2:** Measured Relative End Masses Obtained from the Thermogravimetric Reduction of La_1–*x*_Ca_*x*_MnO_3+δ_ and the Respective δ-Values Calculated from Them Using eq 2

*x*	*m*_e_/*m*_i_	δ-value	3 + δ
0 (N_2_-treated)	0.9621	0.076	3.076
0 (air-treated)	0.9582	0.137	3.137
0.1	0.9562	0.088	3.088
0.2	0.9535	0.047	3.047
0.3	0.9502	0.011	3.011
0.4	0.9439	0.010	3.010
0.5	0.9368	0.011	3.011

From these data, it can be read that the amount of released oxygen increases along with increasing Ca content in the studied materials. These higher mass losses due to oxygen release do not correlate with higher nonstoichiometry. In contrast, higher Ca contents in the materials, although inducing higher mass losses upon reduction, result in smaller nonstoichiometry. This is especially the case for the samples with *x* ≥ 0.3. The reason for this lies in the different decomposition products that are included in the δ-calculation. LaMnO_3+δ_ decomposes to La_2_O_3_ and MnO, whereas La_1–*x*_Ca_*x*_MnO_3+δ_ (*x* = 0.1–0.5) decomposes to La_2_O_3_ and Ca_*x*/(*x*+1)_Mn_1/(*x*+1)_O, as can be seen from Scheme 3 and Figure S2. This also introduces additional stages in the reduction reaction (Figure S3). Oxygen incorporated in La_2_O_3_ and Ca_*x*/(*x*+1)_Mn_1/(*x*+1)_O is not released and therefore does not appear as an absolute mass loss but is considered in the δ-calculations.

Ca substitution for La correspondingly decreases the amount of La_2_O_3_ in the decomposition products. At the same time, an increase in the decomposition product Ca_*x*/(*x*+1)_Mn_1/(*x*+1)_O occurs, which contains less oxygen per metal than La_2_O_3_, resulting in higher absolute oxygen release.

The obtained δ-values in this work are in agreement with the previously reported results. The δ-value of rhombohedral LaMnO_3+δ_ with δ > 0.13 is significantly above the limiting value described in the literature, at which the orthorhombic structure occurs (δ < 0.1).^18,25^ The structural change under a nitrogen atmosphere to an orthorhombic structure happens due to increased amounts of Mn^3+^ compensating for the lower oxygen nonstoichiometry and resulting in Jahn–Teller-distortions, which also was reported by Töpfer et al.^18,24^ La_0.9_Ca_0.1_MnO_3+δ_ exists in a less-distorted, orthorhombic structure (O). The literature describes this structure to be present at 0.077 < δ < 0.159.^12^ The observed value of δ = 0.088 fits well with the literature values. For the samples *x* ≥ 0.3, the orthorhombic O structure and δ-values near zero have been also observed by Dabrowski et al.^12^

### Thermogravimetric Experiments on La_1–*x*_Ca_*x*_MnO_3+δ_ as a Function of Atmosphere (O_2_, Air, and N_2_)

In this section, we investigated the changes in oxygen nonstoichiometry within the La_1–*x*_Ca_*x*_MnO_3+δ_ series as a function of atmosphere and temperature. TGA of La_1–*x*_Ca_*x*_MnO_3+δ_ (*x* = 0–0.5), calcined at 1100 °C in air, was performed at 950 °C in synthetic air (80% N_2_, 20% O_2_), O_2_, and N_2_ atmosphere. Always, two cycles were measured.

**Figure 4:**
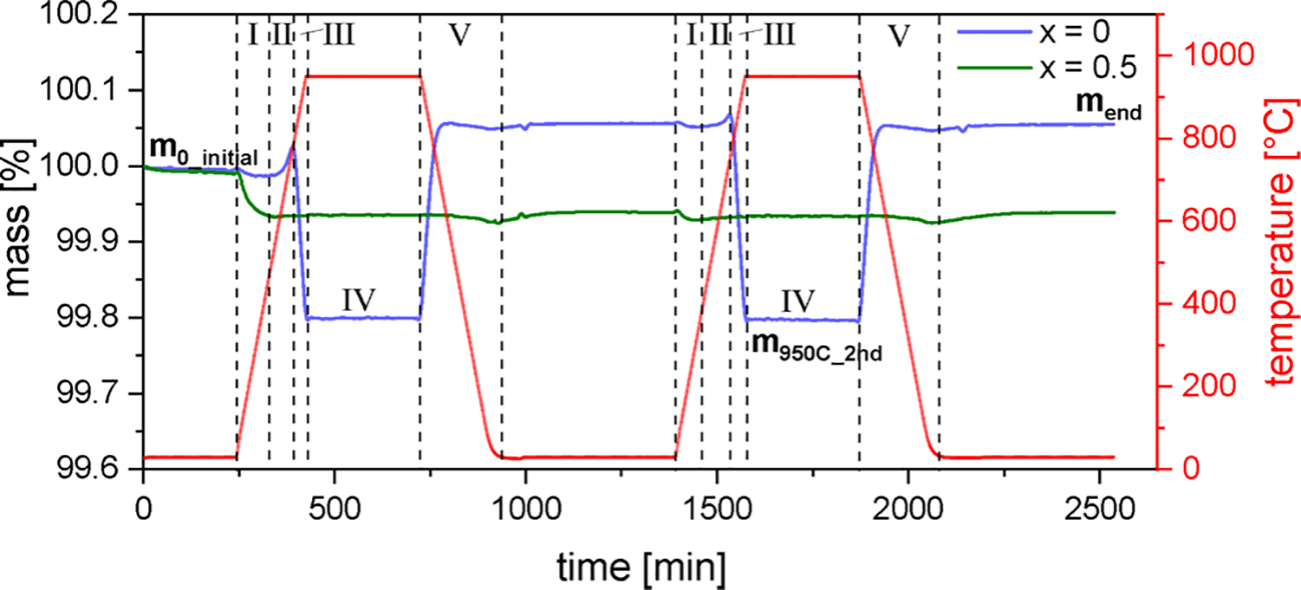
Smoothed TGA data of LaMnO_3+δ_ and La_0.5_Ca_0.5_MnO_3+δ_ measured in an oxygen atmosphere. Heating and cooling were performed with a rate of 5 K/min, and the holding time at 950 °C was set to 5 h. For each measurement, two cycles were performed, where cycle 1 serves for investigation of an atmospheric change and cycle 2 serves for reversibility. *m*_0,initial_, *m*_950°C,2nd_, and *m*_end_ mark the regions from which masses were extracted for the calculation of the samples’ δ-value changes. Also displayed are the regions I–V, in which mass changes are observed and which are discussed in detail in the text.

Figure 4 shows the results of two samples (*x* = 0 and 0.5) cycled in a pure oxygen atmosphere. The first of the cycles gives the mass changes along with the heating of the materials, previously synthesized in air, under an oxygen atmosphere. The material is not in the equilibrium state. The second cycle is designed to detect reversible temperature-induced mass changes. Mass increases and decreases are associated with oxygen uptake and release. Different ranges I–V were introduced in order to describe the occurring processes. For *x* = 0, five ranges can be recognized. In range I, below 550 °C, an irreversible mass loss occurs. In range II, between 550 and 750–800 °C, the mass increases. In range III, a reversible mass loss occurs for *T* > 750–800 °C. Range IV describes the dwell time at the maximum temperature applied (950 °C), in which the mass stays constant. On cooling, summarized as range V, the process III reproduces in the reversed form, while the partially irreversible processes I and II may modify the cooling profile but cannot be distinguished clearly.

In the first cycle, the unsubstituted sample, after a slight initial mass decrease in range I, exhibits a mass increase of ≈0.03% in range II. Upon further heating, a mass decrease of about 0.2% occurs (range III). During the dwell time at 950 °C, the mass remains constant (range IV), followed by a mass increase of 0.26% upon cooling. It finally results in an overall mass gain of the room temperature structure. At this elevated oxygen content, the second heating cycle shows a symmetric profile and is leading to the same mass as after cycle 1.

For *x* = 0.5, in the first cycle, only the irreversible mass loss in range I occurs and is much more pronounced (≈0.06%). In the second cycle, a small mass loss in range I is still recognizable, yet the final mass after two cycles is similar to the mass obtained after cycle 1.

**Figure 5:**
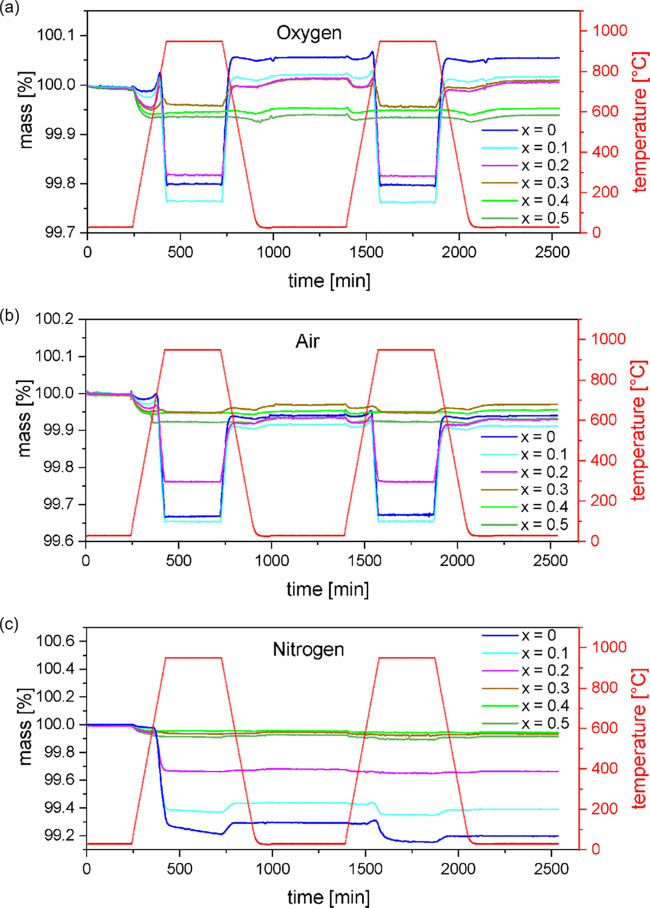
Smoothed TGA data of La_1–*x*_Ca_*x*_MnO_3+δ_ (*x* = 0–0.5) measured in three different atmospheres: (a) oxygen, (b) synthetic air, and (c) nitrogen. Heating and cooling were performed with a rate of 5 K/min, and the holding time at 950 °C was set to 5 h. For each measurement, two cycles were performed, where cycle 1 serves for investigation of an atmospheric change and cycle 2 serves for reversibility.

Figure 5 displays the thermogravimetric measurements of all air-synthesized samples in three different atmospheres (O_2_, synthetic air, and N_2_).

For the measurements in an oxygen atmosphere (Figure 5a), it can be observed that the samples with Ca contents below *x* = 0.3 behave similar to the behavior described above for the unsubstituted sample LaMnO_3+δ_, while the increasingly substituted samples behave similar to La_0.5_Ca_0.5_MnO_3+δ_.

In the first measurement cycle, the slight mass loss in range I becomes stronger with the growing amount of Ca. It is assumed to occur due to not fully equilibrated samples after synthesis, possibly due to the different temperature profiles and the fast cooling rate standing in contrast to the slow equilibration of the samples. Up to *x* = 0.3, this mass loss is followed by a slight increase in range II, which becomes weaker with increasing Ca content. It vanishes for *x* ≥ 0.4. The mass gain suddenly is terminated at around 750 °C at the start of range III. This is caused most likely by a structural change.^31^ The reversible mass change in range III increases from *x* = 0 to *x* = 0.1 and then decreases until it does not occur for *x* ≥ 0.4 anymore. The process behind this is the equilibration of the sample to temperature and atmosphere in the high-temperature regime as described by Mizusaki et al.^20^ and Miyoshi et al.^31^ and only occurs for samples containing excess oxygen.

**Figure 6:**
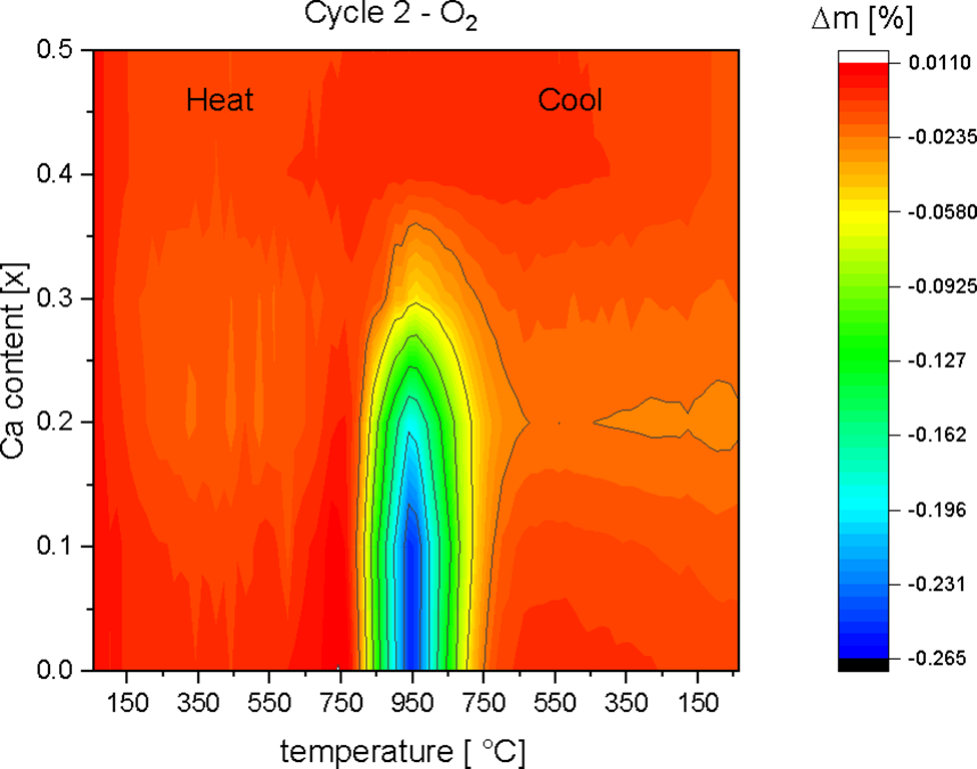
Contour plot of the mass changes occurring for the series La_1–*x*_Ca_*x*_MnO_3+δ_ during the second heating and cooling cycle in an oxygen atmosphere. The temperature range from 100 to 950 °C is shown.

In the second cycle, the mass loss in range I and the mass gain in range II are almost absent. The reversible mass loss in range III has been calculated. As the sample is in equilibrium only after cycle 1, the mass loss from RT (after cycle I) to 950 °C (cycle 2) has been calculated. This mass loss amounts to −0.26, −0.25, and −0.19% for the samples *x* = 0, *x* = 0.1, and *x* = 0.2, respectively. It is much less for *x* = 0.3 and vanishes for *x* = 0.4 and *x* = 0.5. In a contour plot, showing the mass change, the effect of this oxygen release is summarized (Figure 6). The mass difference is calculated from the mass at RT after cycle 1, when the sample is in equilibrium, and from the masses during the heating cycle up to 950 °C (cycle 2). It makes clear that in region III, cycle 2, the oxygen release decreases with the Ca content *x*.

In a synthetic air atmosphere (Figure 5b), the samples’ behavior is comparable, although the extent of mass changes differs as the oxygen partial pressure is reduced and is basically similar to the one during synthesis. An overall mass loss within the room temperature structure can be observed after two cycles for all studied samples. This is in contrast to the behavior of LaMnO_3+δ_ in a pure oxygen atmosphere, for which an overall mass gain was observed. This can be attributed to the process in range III and its reverse process in range V, which does not recover the initial mass. It can be explained by the equilibration in the high-temperature regime, this time under lower oxygen partial pressure.^20,31^

The studied materials behave differently under a nitrogen atmosphere (Figure 5c). In the first cycle, upon heating, a significant mass loss in range III (0.8% for of LaMnO_3+δ_) can be observed for the samples containing up to 20% of calcium and only a slight mass loss (−0.05 to −0.07%) can be observed for higher calcium contents, which can be attributed to range I. The mass increase in range II is not observed for any of the samples as no oxygen is provided. Compared to the initial mass, there is an overall mass loss for all samples after two measurement cycles. It is assumed that in nitrogen, the samples are reduced toward their stoichiometric compositions, leading to a massive release of oxygen for those samples with high oxygen nonstoichiometry δ, but is less pronounced for samples without or very small oxygen nonstoichiometries. LaMnO_3+δ_ and La_0.9_Ca_0.1_O_3+δ_ still lose oxygen in the second cycle, meaning δ may not have reached zero yet.

### Determination of Changes in Oxygen Nonstoichiometry in Various Atmospheres

The mass changes shown in Figure 5 could be further correlated with changes in the oxygen-nonstoichiometry Δδ of the samples according to eq S1. Masses were taken from the three differently named ranges (*m*_0,initial_, *m*_950°C,2nd_, and *m*_end_) in Figure 4.

For all samples, the initial stoichiometry calculated from reduction experiments was taken as a starting point, that is, 100% of the mass (*m*_0,initial_). The mass changes shown in Figure 5 were correlated with changes in the oxygen-nonstoichiometry Δδ of the samples according to eq S1.

**Figure 7:**
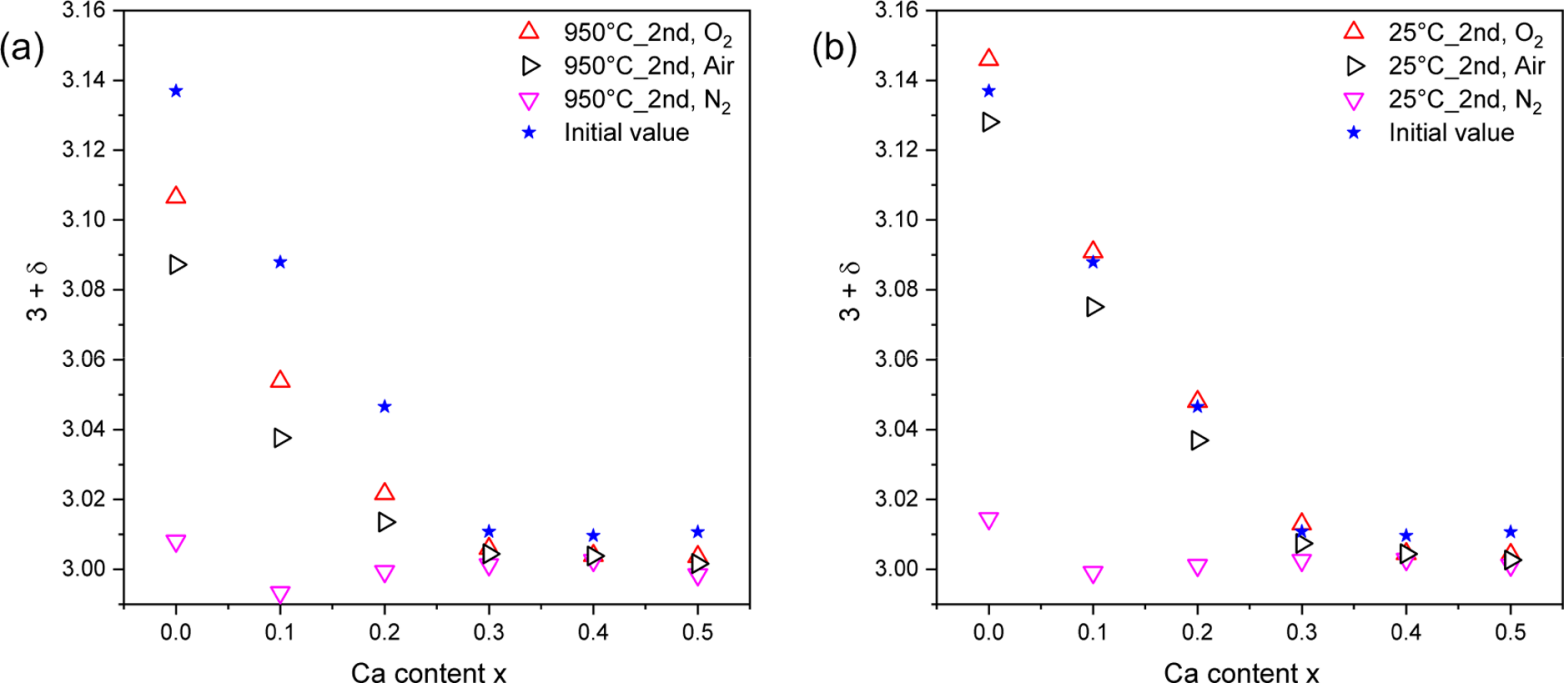
(a) Comparison of the δ-value changes after heating La_1–*x*_Ca_*x*_MnO_3+δ_ (*x* = 0–0.5) twice to 950 °C in three different atmospheres: oxygen (red), air (black), and nitrogen (pink). (b) Comparison of the room temperature materials’ oxygen content after atmospheric changes (air, nitrogen, and oxygen).

The initial oxygen nonstoichiometries are plotted as the blue stars in Figure 7. The behavior with *x* is similar to that reported for La_1–*x*_Sr_*x*_MnO_3+δ_.^20^ The changes are displayed for the different atmospheres at 950 °C in cycle 2 in Figure 7a and at RT after cycle 2 in Figure 7b. All the relative mass changes and the δ-value calculated from these for Figure 7 are listed in Table S2.

In general, heating up to 950 °C (Figure 7a) leads to a decrease in the initial oxygen nonstoichiometry, which can be mainly attributed to range III. The extent strongly depends on the Ca content and atmosphere. For small Ca contents (*x* = 0–0.2), significant δ-value changes can be observed. For high Ca contents (*x* = 0.3–0.5), the atmospheric and temperature effects are very small and hardly distinguishable, as the initial oxygen stoichiometry is close to 3.

The temperature-related decrease in the oxygen nonstoichiometry at 950 °C for samples with small Ca contents (0 ≤ *x* ≤ 0.2) could be attributed to the structural change at 750 °C and the following equilibration to temperature and atmosphere in the range III.^20,31^ For the other samples (*x* = 0.3–0.5), the observed changes can simply be attributed to the irreversible mass loss during the first heating ramp below 550 °C (range I).

The extent of these temperature-related effects differs depending on the applied atmosphere. Taking the initial sample nonstoichiometry as a basis, the smallest changes can be observed when the samples are heated to 950 °C in an oxygen atmosphere. The biggest δ-value changes are visible for *x* = 0.1, with δ decreasing from 0.088 to 0.053 (Δδ = 0.035).

In air, a change by Δδ = 0.05 (for both *x* = 0 and *x* = 0.1) can be observed, which was expected due to the decreased oxygen partial pressure in air. This dependence on oxygen partial pressure at temperatures above 700 °C was reported by Miyoshi et al.^31^ and Mizusaki et al.^20^ The biggest decrease in the δ-value can be seen under a nitrogen atmosphere (with Δδ = 0.13 and 0.10 for *x* = 0 and 0.1, respectively). This can be attributed to the oxygen partial pressure being drastically reduced, leading to final 3 + δ close to the stoichiometric value of 3, as reported by Mizusaki et al.^20^ for La_(1–*x*)_Sr_*x*_MnO_3+δ_ (LSM).

Thermogravimetric measurements after cooling the samples back to room temperature (Figure 7b) show that the samples do not only undergo the reported temperature-related changes but also atmosphere-related changes in the room temperature materials occur. Changing the atmosphere from air to oxygen, the synthetic air or nitrogen atmosphere has an influence on the oxygen nonstoichiometry. For materials with calcium contents higher than *x* = 0.3, the δ-value decreases irreversibly in all atmospheres and remains constant afterward. For lower calcium contents, the δ-value increases in an oxygen atmosphere, as more oxygen is available than during synthesis. Under the air and nitrogen atmosphere, a decrease in oxygen nonstoichiometry has been observed. No change is expected in the air atmosphere, as the sample was synthesized in air. As can be seen from Figure 5b, while heating up, all samples undergo an irreversible mass loss in range I. We attribute this to a different temperature profile during calcination and the present measurement. In a nitrogen atmosphere, δ decreases toward zero, meaning the samples are almost stoichiometric afterward. Only for pure LaMnO_3+δ_, δ remains 0.01, which might be due to slow oxygen release rates at low oxygen partial pressures and 950 °C. For materials with small contents of calcium, it is possible to vary the oxygen nonstoichiometry with a changing atmosphere. This is in agreement with the δ/*x* phase diagram published by Dabrowski et al.,^12^ which shows less susceptibility to changes in δ for samples with higher *x*.

### Thermogravimetric Analysis of La_0.9_Ca_0.1_MnO_3+δ_ in Dependence on the Maximum Cycling Temperature

**Figure 8:**
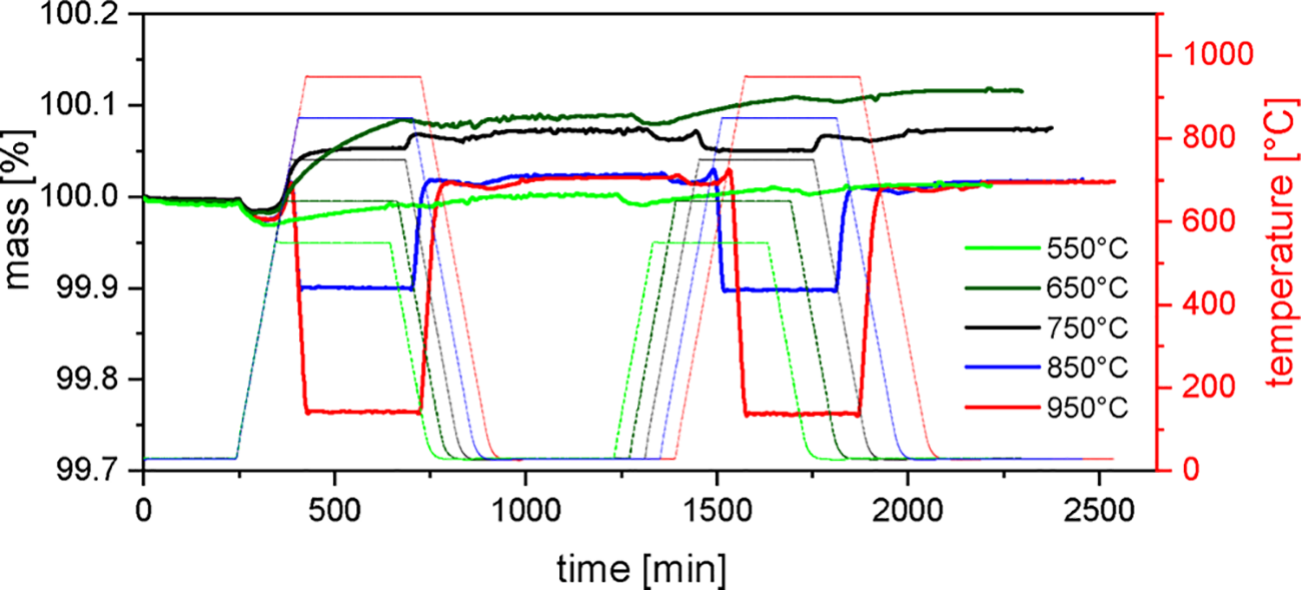
Smoothed TGA data of La_0.9_Ca_0.1_MnO_3+δ_ heated twice to 550, 650, 750, 850, and 950 °C in an oxygen atmosphere. The initial structure is always the air-calcined sample. By this, the mass changes in dependence of the temperature should be determined.

To clarify, in how far the sample *x* = 0.1 is in equilibrium at various temperatures during the previous heating experiment to 950 °C, La_0.9_Ca_0.1_MnO_3+δ_ was investigated thermogravimetrically with a similar profile, but this time, the maximum temperature was varied in 100 °C steps from 550 to 950 °C. For each temperature, two cycles were measured in an oxygen atmosphere, with a dwell time of 5 h at the respective temperatures (Figure 8). The initial structure is always the air-calcined sample.

It can be observed that up to *T* ∼ 750 °C, an irreversible oxygen uptake occurs, reflected by a mass increase, which remains present after cooling to room temperature. The highest mass increase can be observed at 650 °C. Heating to 850 °C and higher leads to a rapid mass loss and oxygen release at 750 °C, which is taken up reversibly upon cooling. This behavior can also be observed qualitatively in the second cycle at 750 °C and was discussed before as a reversible structural change occurring at around 750 °C.^31^

The results show that the processes below 750 °C are slow, as expected for lower temperature. The equilibration takes a long time and is still not completed under the presented conditions. This applies especially for 650 °C, which is also the temperature where the highest mass gain is achieved. This and the termination of the oxygen uptake at around 750 °C are hints for a structural change occurring at *T* > 750 °C.^20,31^

**Figure 9:**
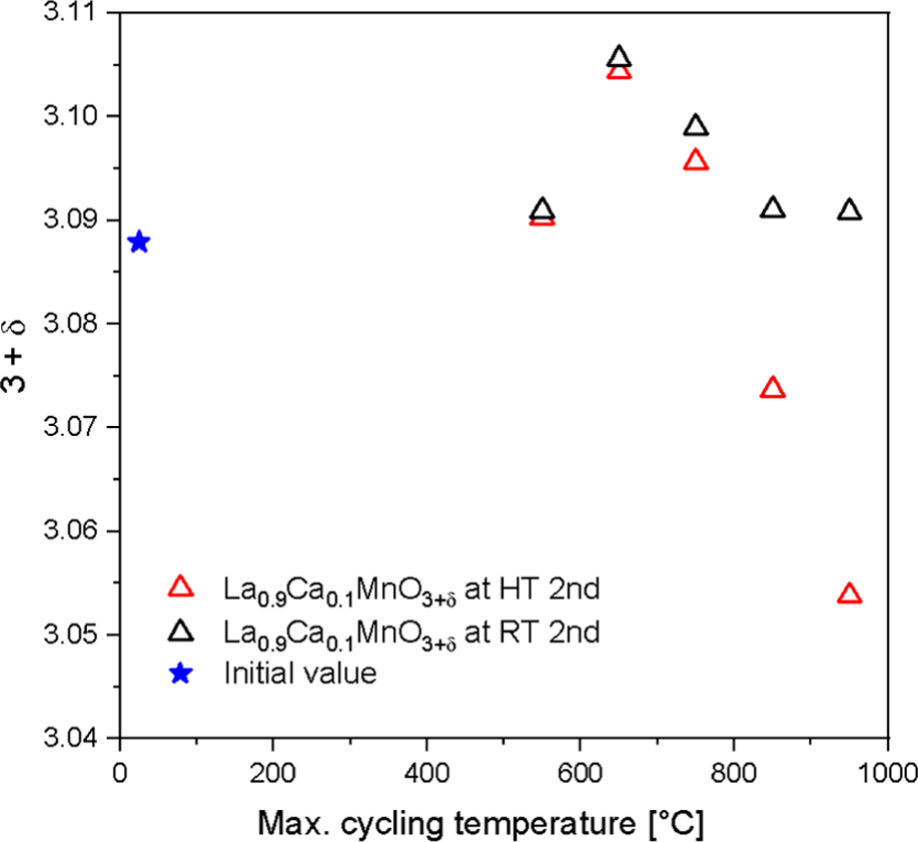
3+δ-value of La_0.9_Ca_0.1_MnO_3+δ_ in an oxygen atmosphere during the second cycle at 550, 650, 750, 850, and 950 °C (red triangles) and after subsequent cooling to room temperature (black triangles). As a comparison, the initial state at room temperature (blue stars) is shown.

Figure 9 shows the corresponding oxygen nonstoichiometry calculated from the mass changes seen in Figure 8, during the second cycle at elevated temperatures (red triangles) and the residual change after cooling down to room temperature (black triangles). Thus, when the sample La_0.9_Ca_0.1_MnO_3+δ_ is heated to 650 °C, a maximum in oxygen uptake with Δδ = 0.02 can be observed for both temperatures (650 °C and RT) as it is an irreversible change. Up to a temperature of 750 °C, the incorporated oxygen is contained in the structure when the sample is cooled down. In both cases, the oxygen uptake can be attributed to range II. From the TGA curves in Figure 8, it can be inferred that the oxygen uptake is a slow process. When heated to temperatures higher than 750 °C, the sample equilibrates with the temperature and atmosphere as described above. The amount of excess oxygen decreases with *T*.^20^ Compared to the initial room temperature value, the δ-value after two cycles is essentially identical.

### Thermogravimetric Study of Rhombohedral and Orthorhombic LaMnO_3+δ_

**Figure 10:**
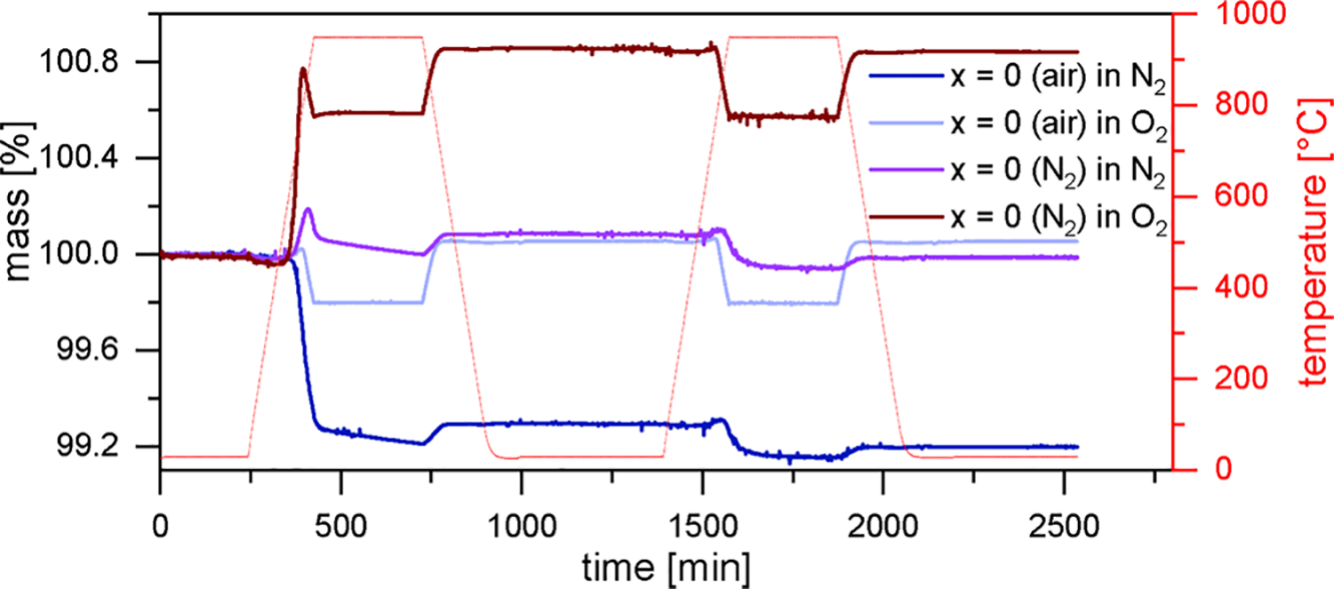
Thermogravimetric investigation of variously synthesized LaMnO_3+δ_ in O_2_ and N_2_ atmospheres. Heating and cooling were performed with a rate of 5 K/min, and the holding time at 950 °C was set to 5 h. For each measurement, two cycles were performed.

The rhombohedral, air-synthesized LaMnO_3+δ_ (δ = 0.137) was tempered for 4 h at 1100 °C in a N_2_ atmosphere. The structure changed to the highly distorted orthorhombic O′ modification, as could be confirmed with PXRD and as has been previously reported in the literature.^16^ Relating this to the δ-value of 0.076 as measured by thermogravimetric decomposition, this is in agreement with most of the previous studies.^16,18,21^ In contrast, Dabrowski et al.^12^ have reported the less distorted O structure for this δ-value. Thermal behavior of both polymorphic modifications under two different atmospheres (O_2_ and N_2_) was compared using TGA, and the measurements are displayed in Figure 10. Up to 800 °C under an oxygen atmosphere, the N_2_-tempered sample, which is present in the orthorhombic modification, takes up oxygen strongly, leading to a mass change of about Δ*m* = 0.8%. This mass change is much stronger than that observed for the in-air-synthesized sample (Δ*m* = 0.03%). In the oxygen atmosphere, the previously described mass loss due to an expected structural change is visible at around 750–800 °C and with a similar decrease (Δ*m* = ±0.22%) as observed for the in-air-synthesized sample. Under the nitrogen atmosphere, slight oxygen uptake and release occurs to some amount, which might be explained by slow equilibration, but the end mass is very close to the initial one.

The rhombohedral structure of the air-synthesized sample heated up in a nitrogen atmosphere, which has been described before, does not show oxygen uptake during the first heating cycle. Starting at around 700 °C, it releases a large amount of oxygen upon heating under a nitrogen atmosphere. It can be seen that with the choice of the atmosphere and temperature profile, the state of the sample can be changed. This shows that differently synthesized or treated samples (air and N_2_), starting with different δ-values (δ = 0.137 vs δ = 0.076), are in the same state after exposure to the oxygen atmosphere at temperatures above 750 °C. The sample with a lower δ-value at the start takes up significantly higher amounts of oxygen compared to the one with a higher δ-value. In a nitrogen atmosphere, the sample with a higher δ-value correspondingly releases much more oxygen than the sample with lower initial δ.

### Calculation of the Mn-Oxidation State in the La_1–*x*_Ca_*x*_MnO_3+δ_ Series Using Thermogravimetric Analysis

**Figure 11:**
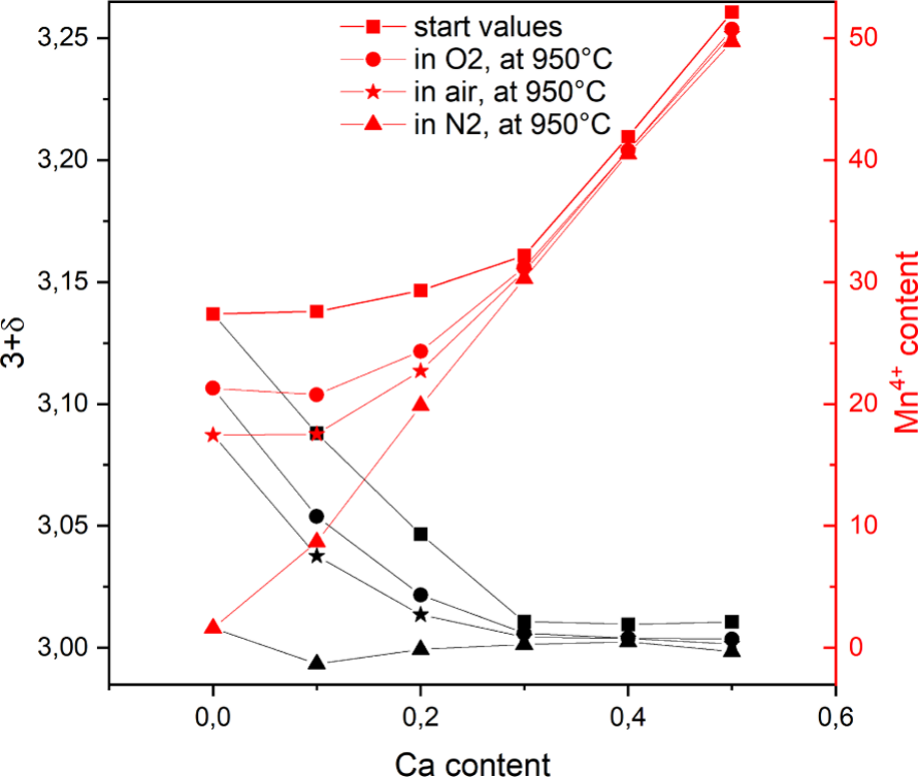
Mn^4+^ content within La_1–*x*_Ca_*x*_MnO_3+δ_ (*x* = 0–0.5) calculated from δ-values determined by TGA.

If the δ-value is known, the Mn-oxidation states can be calculated from Scheme 2, where the Mn^4+^ content mainly corresponds to 2δ + *x*. In general, the defect chemistry of La_1–*x*_Ca_*x*_MnO_3+δ_ can be separated into intrinsic and extrinsic effects, leading to a change in the Mn-oxidation state. The content of Mn^4+^ within the samples can be influenced by either the change of the oxygen content within the sample as a reaction to temperature and atmosphere, which we call intrinsic, or by the introduction of Ca^2+^, which we call extrinsic.^20,35^ Hence, for the unsubstituted LaMnO_3+δ_, the Mn-oxidation state is solely intrinsically determined as a reaction of the sample with the surrounding atmosphere. For low amounts of Ca substitution, the Mn oxidation state is influenced intrinsically, by its reaction with the atmosphere, and extrinsically, by Ca substitution. At high Ca contents, the extrinsic effect dominates and the Mn oxidation is mainly influenced by cation replacement. When looking at the Mn^4+^ content within the room temperature structures, shown as red squares in Figure 11, it can be seen that LaMnO_3+δ_ (*x* = 0) contains ∼26% of Mn^4+^ (2δ). Heating to 950 °C in various atmospheres (Figure 5) decreases the Mn^4+^ concentration to about 2% (in N_2_). The Mn^4+^ concentration is highly dependent on the atmosphere and the corresponding oxygen partial pressure. The described intrinsic effect can be clearly defined here, as the effects cannot be attributed to Ca substitution.

The substitution of La by increasing amounts of Ca is accompanied by a general increase in the Mn^4+^ content. Similar to the unsubstituted sample, the Mn-oxidation state decreases with decreasing oxygen partial pressure for low Ca contents (*x* = 0–0.2). The extent of Mn^4+^ changes becomes smaller with increasing Ca content. For high Ca contents (*x* = 0.3–0.5), the increase in Mn^4+^ is independent of the atmosphere and scales almost linearly with *x*.

In general, the lowest Mn^4+^ contents are achieved in the nitrogen atmosphere, where the Mn-oxidation state increases almost perfectly linearly up to around 50% Mn^4+^ for *x* = 0.5 over the whole sample series (*x* = 0–0.5). In the nitrogen atmosphere, most of the intrinsic effects due to reaction with oxygen in the surrounding atmosphere are absent. Extrinsic effects dominate, which can be validated by the almost linear increase in the Mn^4+^ content in N_2_, which is nearly perfectly scaling with the Ca-content *x*. Deviations show contributions from intrinsic effects due to reaction with surrounding oxygen. These deviations should scale with the oxygen partial pressure and can be observed for low Ca contents up to *x* = 0.2. Therefore, it is assumed that the Mn oxidation in this range is influenced by intrinsic and extrinsic effects. For higher Ca contents (*x* = 0.3–0.5), only extrinsic effects are visible.

### Investigation of the Mn Electronic State Using XANES

**Figure 12:**
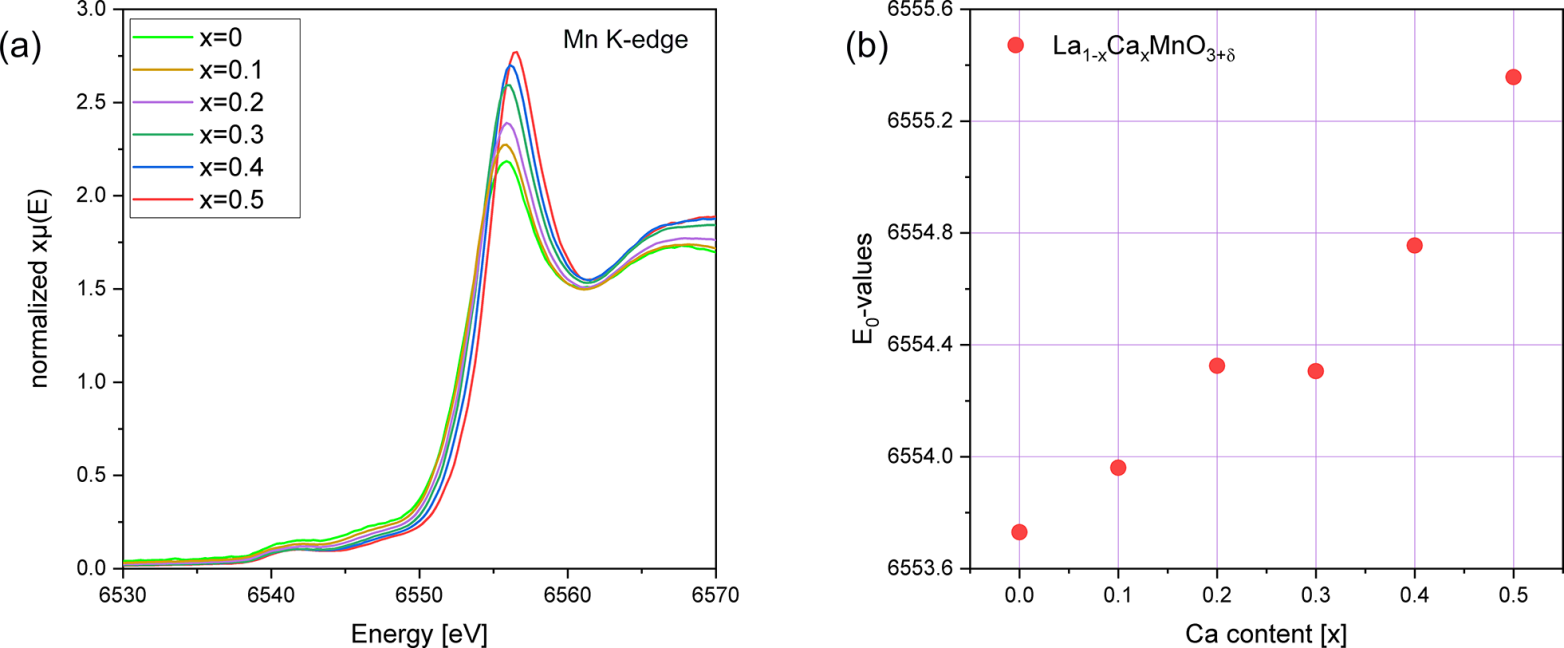
Normalized XANES Mn K-edge spectra of air-synthesized La_1–*x*_Ca_*x*_MnO_3+δ_ (*x* = 0–0.5). The unsubstituted LaMnO_3+δ_ is rhombohedral (*R*3̅*c*), while all Ca-substituted samples are of the orthorhombic (*Pnma*) structure. (b) Calculation of the edge positions, *E*_0_, for the samples *x* = 0–0.5 that are presented in (a). The value *E*_0_ correlates with the electronic state of Mn.

To provide experimental evidence on the electronic state of the Mn cations, XANES experiments were performed in both the hard and soft X-ray regimes. The near-edge region of the normalized Mn K-edges from air-sintered La_1–*x*_Ca_*x*_MnO_3+δ_ (*x* = 0–0.5) is shown in Figure 12a. For the air-synthesized samples, including rhombohedral *x* = 0, it can be observed that the Mn K-edge shifts to higher energies with increasing Ca content. This shift can be attributed to a change in the formal oxidation state of Mn, namely, an increased Mn^4+^ content, within the sample.^36^Figure 12b shows the edge positions, *E*_0_, here defined as the value at half edge rise, as a function of the Ca content (*x*). It can be observed that between *x* = 0.2 and *x* = 0.3, a change in the electronic state occurs. This fits the results previously obtained in the TGA, where a change in defect chemistry behavior was deduced between *x* = 0.2 and *x* = 0.3. Compositions of the La_1–*x*_Ca_*x*_MnO_3+δ_ series up to *x* = 0.2 show again that Mn oxidation is influenced by both extrinsic and intrinsic effects, while only extrinsic effects are present in samples with higher Ca contents (*x* = 0.3–0.5). The hard X-ray XANES results, shown in Figure 12, agree with these assumptions.

**Figure 13:**
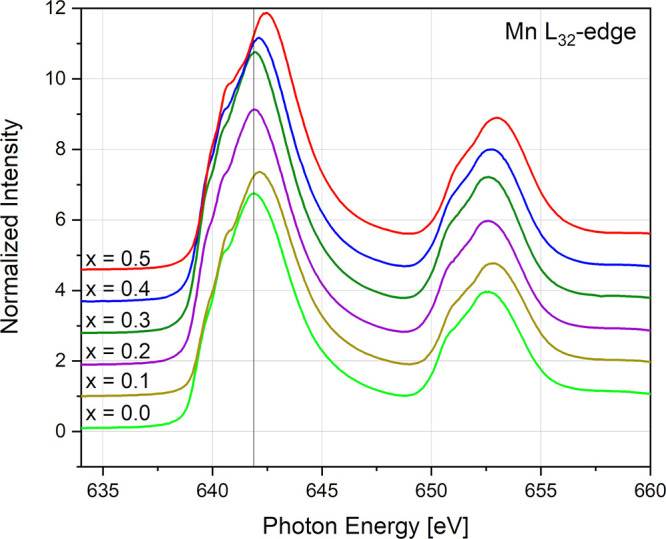
Normalized Mn L_32_-edge spectra of the La_1–*x*_Ca_*x*_-MnO_3+δ_ (*x* = 0–0.5) series.

However, further quantification of these changes is difficult, as octahedral distortions due to a Jahn–Teller-effect, occurring preferably in low Ca regimes, bias the edge positions. Ramos et al.^37^ found for LaMnO_3_ that a shift in the K-edge position might occur due to a contraction of the long Mn–O bonds even if there is no change in the Mn-oxidation state.

**Figure 14:**
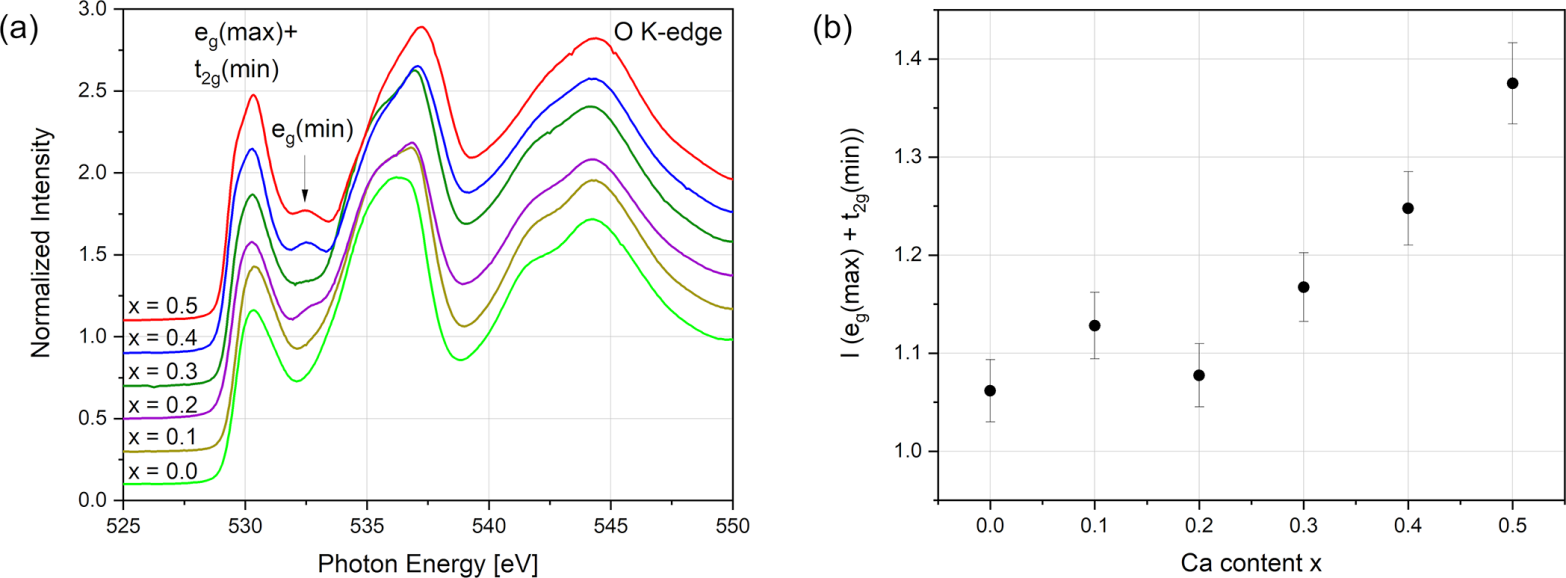
(a) Normalized O K-edge spectra of the La_1–*x*_Ca_*x*_MnO_3+δ_ (*x* = 0–0.5) series. (b) Normalized intensity of the e_g_(max) + t_2g_(min) peak plotted against the Ca content within the La_1–*x*_Ca_*x*_MnO_3+δ_ (*x* = 0–0.5) series. Error bars were estimated from uncertainties in the normalization procedure.

In Figure 13, the Mn L_32_-edge spectra are displayed, which show a behavior similar to the La_1–*x*_Sr_*x*_MnO_3−δ_ (LSM) material system,^38^ where the position of the L_3_ white line shifts to higher energies with increasing Sr content. The O K-edge (Figure 14a), in turn, is also similar to what was observed for LSM:^38^ with increasing substitution with the divalent A-site cation, minority e_g_-states emerge at ∼533 eV. This is explained by smaller overlap of this energy region with La-derived electronic states (La 5d–O 2p-hybridized states) at 535 eV, making a clear distinction possible. Most revealing, although, is the leading edge, which consists of overlapping e_g_ majority and t_2g_ minority states formed by O 2p–Mn 3d hybridization. These states are, especially for the Ca lean samples, indistinguishable because exchange splitting Δ_ex_ and crystal-field splitting 10*Dq* are of similar magnitude as it is the case in the LSM system.^38^ The intensity of this signal e_g_(max) + t_2g_(min) is a direct measure of the hole concentration^38^ and thus can be used to assess the Mn^3+^/Mn^4+^ concentration semiquantitatively. Here also, the intensity (can be approximated by the peak height, as only small variations in peak width and shape are observed) as a function of Ca content (Figure 14b) indicates the same change in defect chemistry as TGA and hard X-ray XANES between *x* = 0.2 and 0.3. Additionally, for high Ca substitutions, (*x* ≥ 0.3) e_g_(max) + t_2g_(min) is split, which can be explained by changes in the electronic structure and ligand field, so that the values of Δ_ex_ and 10*Dq* are of different magnitudes, and e_g_(max) and t_2g_(min) are distinguishable again.

## Conclusions

La_1–*x*_Ca_*x*_MnO_3+δ_ (*x* = 0–0.5) was synthesized in an air atmosphere using solid-state synthesis techniques. The structure of LaMnO_3+δ_ was found to be dependent on the atmosphere and changed from a rhombohedral modification in air to the strongly Jahn–Teller distorted orthorhombic polymorph after exposure to the nitrogen atmosphere. The Ca-substituted compositions were all orthorhombic without Jahn–Teller distortion. The lattice parameters decreased with the Ca content, as well as the cell volumes, which was mainly explained by the increase in Mn^4+^ content, being smaller than Mn^3+^ and an increase in divalent Ca^2+^, which is smaller than La^3+^.

TGA of La_1–*x*_Ca_*x*_MnO_3+δ_ in a strongly reducing atmosphere allowed the determination of the initial oxygen nonstoichiometry δ, which decreases with increasing Ca contents. With this calibrated initial nonstoichiometry, significant δ-value changes were observed for small Ca contents (*x* = 0–0.2) in further thermogravimetric experiments performed in various atmospheres (O_2_, air, and N_2_). Higher Ca contents (*x* > 0.3) did not show significant changes in their δ-values, independent of the applied atmosphere. In the TGA experiments, three processes were observed during heating. A slow irreversible loss appears independent of the gas atmosphere at temperatures below 550 °C (in range I), weak for *x* = 0 and becoming stronger with *x*. Above 550 °C, a slow irreversible oxygen uptake becomes active up to 750 °C (range II), which is the strongest for *x* = 0 and disappears for *x* = 0.4. This process is absent under the nitrogen atmosphere. At temperatures higher than 750–800 °C (range III), the samples with *x* ≤ 0.3 equilibrate and nonstoichiometry decreases with *T*, while the extent depends on *p*(O_2_).

The Mn-oxidation state was calculated from the δ-values determined by TGA, assuming the formation of equal A- and B-site vacancies. The influence on the Mn oxidation within the sample series was subdivided into three regions. In pure LaMnO_3+δ_, Mn oxidation was attributed solely to the intrinsic effects, and the oxygen nonstoichiometry is only adapting to the surrounding atmosphere. Low calcium contents up to *x* = 0.2 always contain some amount of Mn^4+^ extrinsically determined by the Ca-content and an additional intrinsic reaction with the atmosphere. In a range of 0.3 ≤ *x* ≤ 0.5, the Mn-oxidation state mainly is extrinsically influenced by the Ca substitution, and the samples show almost no reaction with the atmosphere. In the N_2_ atmosphere, the oxygen nonstoichiometry was reduced to 3 and the Mn^4+^ content scales nearly perfectly with the Ca-content *x*.

Soft and hard X-ray XANES experiments were performed for the series (*x* = 0–0.5) to provide experimental evidence on Mn-oxidation state calculations. A shift of the Mn K-edge toward higher energies was observed with increasing Ca content. This shift was attributed to an increasing change in the formal oxidation state of Mn. From Mn L-edge and O K-edge spectra, similar results could be observed. Between *x* = 0.2 and *x* = 0.3, a change in the electronic state of Mn was detected. It is assumed that at this point, the main influence on the Mn-oxidation state changes from intrinsic effects in *x* = 0–0.2 toward extrinsic effects for *x* ≥ 0.3.

The TGA experiments show that for samples with low Ca content, the oxygen nonstoichiometry 3 + δ and therefore Mn^4+^ content react quickly and reversibly above 750 °C. This susceptibility is decreasing with *x*, but the increasing Ca substitution stabilizes the structure. For low Ca contents, the operation temperature and atmosphere are expected to play an important role. Exposure at temperatures below 750 °C enables one, to some extent, to irreversibly change the oxygen nonstoichiometry by the control of temperature and gas atmosphere. Knowledge of the defect chemistry obtained from the results of this study can be applied for design and development of a suitable air electrode material for solid oxide cell operation. Our results show that the choice of operation temperature, below or above 750 °C, is expected to show a different behavior, as the reaction of the material with the atmosphere changes.

## Supporting Information Available

PXRD measurements of La_1–*x*_Ca_*x*_MnO_3+δ_ in three different ranges for accurate structure refinement; comparison of *x* = 0 synthesized in air and additionally tempered in N_2_; calculated lattice parameters and unit cell volumes of La_1–*x*_Ca_*x*_MnO_3+δ_; PXRD patterns of the decomposition products of the La_1–*x*_Ca_*x*_MnO_3+δ_ series; thermogravimetric reduction of air-synthesized La_1–*x*_Ca_*x*_MnO_3+δ_ samples; formula used to calculate δ-value changes within TGA experiments; and calculated δ-value changes from TGA experiments (PDF)
