# Supplementary material for: Oxygen Nonstoichiometry and Valence State of Manganese in La1–xCaxMnO3+δ
Source: ACS Omega. 2021 Apr 2;6(14):9638–52. doi: 10.1021/acsomega.1c00208 (PMC8047706; doi:10.1021/acsomega.1c00208)
Supplement: Supplementary file 1 — ao1c00208_si_001.pdf [file ao1c00208_si_001.pdf]

SUPPORTING INFORMATION

Oxygen Nonstoichiometry and Valence State of Manganese in $La_{1-x}Ca_xMnO_{3+\delta}$

Sabrina A. Heuer[1,2], Roland Schierholz*[1], Evgeny V. Alekseev[1], Lars Peters[3], David N. Mueller[4], Tomáš Duchoň[4], Vaibhav Vibhu[1], Hermann Tempel[1], Lambertus G. J. de Haart[1], Hans Kungl[1] and Rüdiger-A. Eichel[1,2]

[1] Forschungszentrum Jülich GmbH, Institute of Energy and Climate Research (IEK-9), Wilhelm-Johnen-Straße, DE-52425 Jülich, Germany

[2] RWTH Aachen University, Institute of Physical Chemistry, Landoltweg 2, DE-52074 Aachen, Germany

[3] RWTH Aachen University, Institute of Crystallography, Jägerstraße 17-19, DE-52066 Aachen, Germany

[4] Forschungszentrum Jülich GmbH, Peter Gruenberg Institute (PGI-6), Wilhelm-Johnen-Straße, DE-52425 Jülich, Germany

KEYWORDS: Oxygen-nonstoichiometry, Defect chemistry, $LaMnO_3$, Thermogravimetry, XANES.

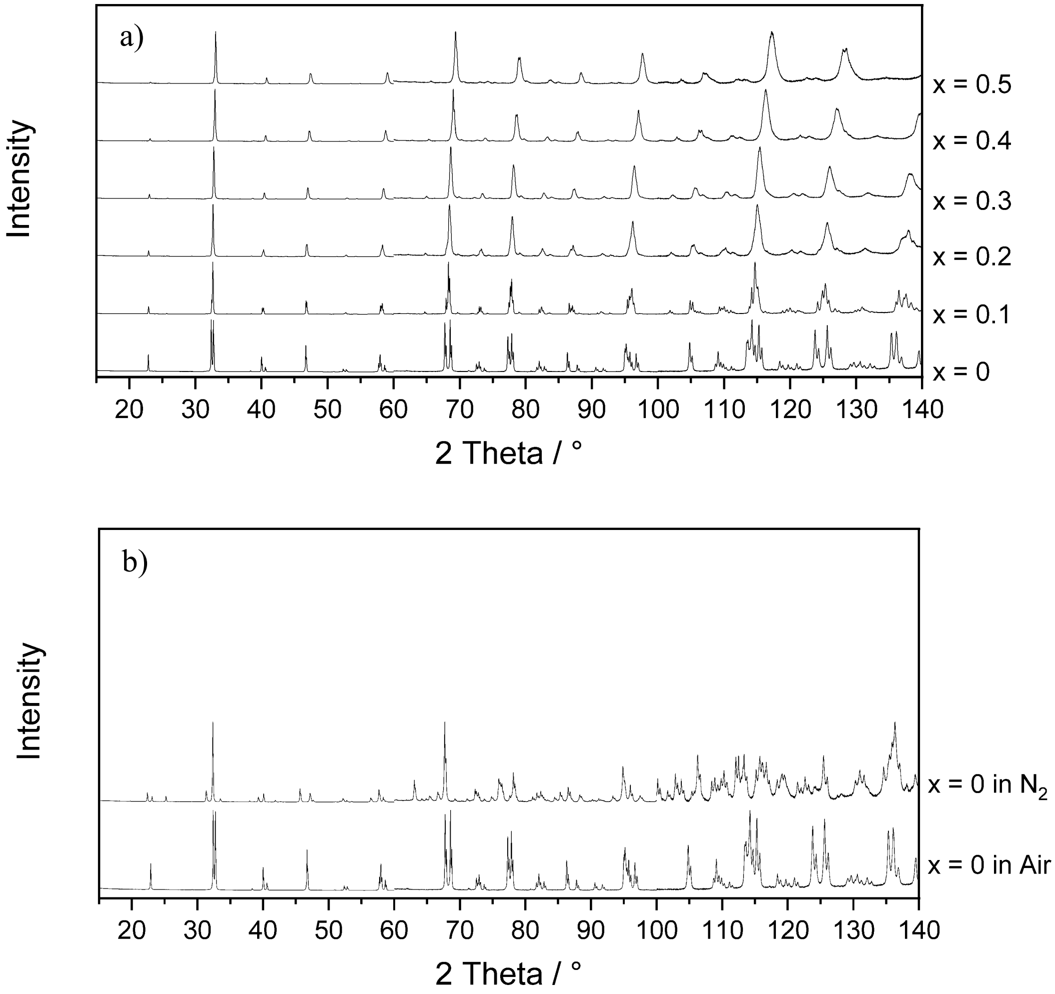

Figure S1: (a) PXRD patterns of Ca-substituted $La_{1-x}Ca_xMnO_{3+\delta}$ synthesized in air, measured in three separate ranges and (b) PXRD patterns of $LaMnO_3$ and $La_{0.9}Ca_{0.1}MnO_{3+\delta}$ prepared in air and additionally tempered in N_2.

Table S1: Determination of structure, lattice parameters (*a*, *b* and *c*) and unit cell volume of air-synthesized $La_{1-x}Ca_xMnO_{3+\delta}$.

x	Structure*	*a* (Å)	*b* (Å)	$b/\sqrt{2}$ (Å)	*c* (Å)	α (°)	V_{unit} (Å³)
0 (air)	$R_{trigonal}$	5.47310(2)	5.47310(2)		5.47310(2)	60.69	
	$R_{hexagonal}$	5.52961(2)	5.52961(2)		13.33640(5)		58.8582(4)
0 (N_2)	O‘	5.70438(4)	7.70664(6)	5.44942(4)	5.53779(5)		60.8625(9)
0.1	O	5.48071(3)	7.75459(4)	5.48332(3)	5.51694(2)		58.6183(5)
0.2	O	5.47266(7)	7.74137(9)	5.47398(6)	5.50091(6)		58.263(2)
0.3	O	5.46811(7)	7.72917(9)	5.46535(7)	5.48332(6)		57.937(2)
0.4	O	5.44308(6)	7.68524(7)	5.43428(5)	5.45876(5)		57.087(1)
0.5	O	5.41906(7)	7.64203(8)	5.40373(6)	5.43157(7)		56.234(2)

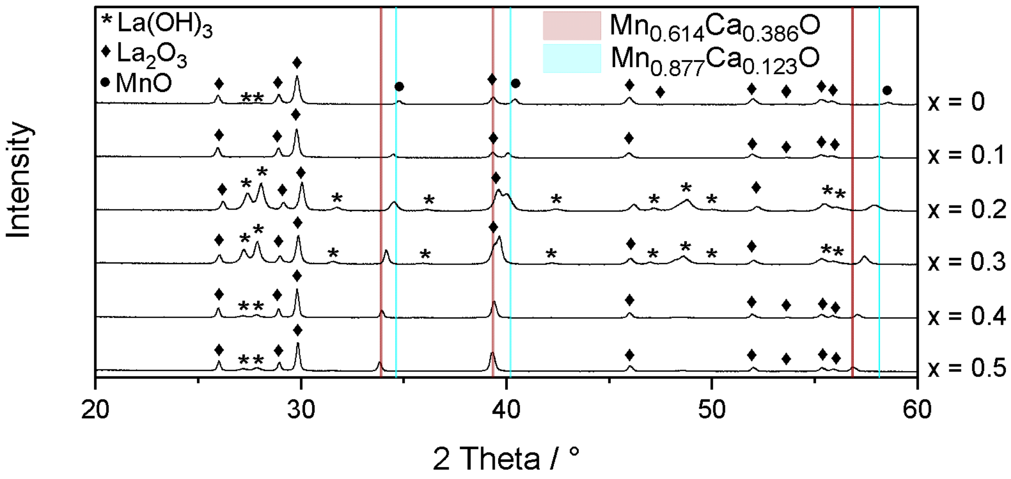

Figure S2: PXRD measurements of the decomposition products of the $La_{1-x}Ca_xMnO_{3+\delta}$ series after reduction in 4%Ar/H_2-atmosphere upon thermogravimetric analysis.

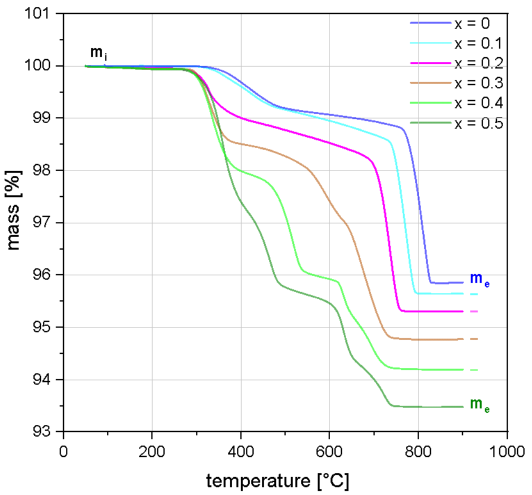

Figure S3: Reduction of the air-synthesized $La_{1-x}Ca_xMnO_{3+\delta}$ ($x = 0 - 0.5$) in Ar/4%H_2 atmosphere. The samples are heated to 900 °C in a reducing atmosphere, where they decompose into their specific products and oxygen. Oxygen release is assumed to cause the obtained mass difference used to determine the δ-value of the samples $La_{1-x}Ca_xMnO_{3+\delta}$.

$$\Delta\delta_{TGA} = -\frac{M_{LCMO}+\delta\cdot M_O-\frac{m_e}{m_i}\cdot(M_{LCMO}+\delta\cdot M_O)}{M_O}$$ Equation S1

Formula used for the calculation of changes in δ-values along with temperature, atmosphere and Ca-content (Table S2). M_{LCMO} is the molar mass of $La_{1-x}Ca_xMnO_{3.00}$ and M_{O} is the molar mass of oxygen. The δ-value represents the initial oxygen non-stoichiometry of the sample. The mass ratio $\frac{m_e}{m_i}$ is the initial sample mass divided by the end mass. The end mass is not specific, it can be chosen as desired. In this work, two mass areas have been chosen as end masses. They are named either m_{950C_2nd} or m_{end}. The respective areas can be found in Figure 5.

Table S2: Calculation of the of the δ-value changes $\Delta\delta$ from the TGA-experiments shown in Figure 4 (i) at 950 °C during the second cycle for Figure 7 (a) (ii) and at RT after the two cycles for Figure 7 (b).

O_2									
x	$\delta_{\mathrm{initial}}$	M_{LCMO} (δ= 0)	3+$\delta_{\mathrm{initial}}$	[m_{950_2nd}/m_i]	$\Delta\delta_{950\_2nd}$	3+δ_{950_2nd}	[m_{e_2nd}/m_i]	$\Delta\delta_{\mathrm{end}}$	3+δ_{end}
0	0.137	241.845	3.137	0.9980	-0.031	3.106	1.0006	0.008	3.145
0.1	0.088	231.962	3.088	0.9976	-0.035	3.053	1.0002	0.003	3.091
0.2	0.047	222.079	3.047	0.9982	-0.026	3.021	1.0001	0.001	3.048
0.3	0.011	212.195	3.011	0.9996	-0.006	3.005	1.0002	0.002	3.013
0.4	0.01	202.312	3.010	0.9996	-0.006	3.004	0.9996	-0.005	3.005
0.5	0.011	192.429	3.011	0.9994	-0.007	3.004	0.9995	-0.007	3.004
air									
x	$\delta_{\mathrm{initial}}$	M_{LCMO} (δ= 0)	3+$\delta_{\mathrm{initial}}$	[m_{950_2nd}/m_i]	$\Delta\delta_{950\_2nd}$	3+δ_{950_2nd}	[m_{e_2nd}/m_i]	$\Delta\delta_{\mathrm{end}}$	3+δ_{end}
0	0.137	241.845	3.137	0.9967	-0.050	3.087	0.9994	-0.009	3.128
0.1	0.088	231.962	3.088	0.9966	-0.050	3.038	0.9991	-0.013	3.075
0.2	0.047	222.079	3.047	0.9976	-0.033	3.014	0.9993	-0.010	3.037
0.3	0.011	212.195	3.011	0.9995	-0.007	3.004	0.9997	-0.003	3.008
0.4	0.01	202.312	3.010	0.9995	-0.006	3.004	0.9996	-0.005	3.005
0.5	0.011	192.429	3.011	0.9992	-0.009	3.002	0.9993	-0.008	3.003
N_2									
x	$\delta_{\mathrm{initial}}$	M_{LCMO} (δ= 0)	3+$\delta_{\mathrm{initial}}$	[m_{950_2nd}/m_i]	$\Delta\delta_{950\_2nd}$	3+δ_{950_2nd}	[m_{e_2nd}/m_i]	$\Delta\delta_{\mathrm{end}}$	3+δ_{end}
0	0.137	241.845	3.137	0.9916	-0.129	3.008	0.9920	-0.122	3.015
0.1	0.088	231.962	3.088	0.9935	-0.095	2.993	0.9939	-0.089	2.999
0.2	0.047	222.079	3.047	0.9966	-0.047	3.000	0.9967	-0.045	3.002
0.3	0.011	212.195	3.011	0.9993	-0.010	3.001	0.9994	-0.008	3.003
0.4	0.01	202.312	3.010	0.9994	-0.007	3.003	0.9995	-0.007	3.003
0.5	0.011	192.429	3.011	0.9990	-0.012	2.999	0.9992	-0.010	3.001
